# LIGHT/LTβR signaling regulates self-renewal and differentiation of hematopoietic and leukemia stem cells

**DOI:** 10.1038/s41467-021-21317-x

**Published:** 2021-02-16

**Authors:** S. S. Höpner, Ana Raykova, R. Radpour, M. A. Amrein, D. Koller, G. M. Baerlocher, C. Riether, A. F. Ochsenbein

**Affiliations:** 1 Department of Medical Oncology, Inselspital, Bern University Hospital, University of Bern, Bern, Switzerland; 2 Department for BioMedical Research, University of Bern, Bern, Switzerland; 3 Department of Hematology and Central Hematology Laboratory, Inselspital, Bern University Hospital, University of Bern, Bern, Switzerland

**Keywords:** Cancer stem cells, Haematopoietic stem cells, Self-renewal

## Abstract

The production of blood cells during steady-state and increased demand depends on the regulation of hematopoietic stem cell (HSC) self-renewal and differentiation. Similarly, the balance between self-renewal and differentiation of leukemia stem cells (LSCs) is crucial in the pathogenesis of leukemia. Here, we document that the TNF receptor superfamily member lymphotoxin-β receptor (LTβR) and its ligand LIGHT regulate quiescence and self-renewal of murine and human HSCs and LSCs. Cell-autonomous LIGHT/LTβR signaling on HSCs reduces cell cycling, promotes symmetric cell division and prevents primitive HSCs from exhaustion in serial re-transplantation experiments and genotoxic stress. LTβR deficiency reduces the numbers of LSCs and prolongs survival in a murine chronic myeloid leukemia (CML) model. Similarly, LIGHT/LTβR signaling in human G-CSF mobilized HSCs and human LSCs results in increased colony forming capacity in vitro. Thus, our results define LIGHT/LTβR signaling as an important pathway in the regulation of the self-renewal of HSCs and LSCs.

## Abstract (web summary)

Regulation of self-renewal is critical during steady state and stress in haematpoietic stem cells (HSCs), and underlies leukaemia pathology. Here, the authors show that LIGHT and its receptor LTβR promote quiescence and self-renewal of HSCs and that LTβR deficiency promotes survival in a mouse leukaemia model.

## Introduction

Hematopoietic stem cells (HSCs) represent a small heterogeneous and hierarchically organized population within lineage negative (Lin^−^) cells^1^. The hierarchy of HSCs is characterized by the progressive decrease of the self-renewal capability from long-term-HSCs (LT-HSCs) over short-term HSCs (ST-HSCs) to multipotent progenitors (MPPs). HSC self-renewal and differentiation is regulated by cell-intrinsic mechanisms such as transcription factors (SATB1, PU-1), cell cycle regulators (CDKN1A, GFI-1), or transcriptional regulators (MSI2)^2–4^. In addition, cell-extrinsic cues from bone marrow (BM) niche cells regulate HSC activity, that is, niche cells secrete stem cell factor (SCF), angiopoietin-1, and thrombopoietin^5^, and express adhesion molecules such as vascular cell adhesion protein 1 (ref. ^6^). In addition, stromal cell-derived factor 1 (CXCL12) promotes the retention of HSCs in the BM and contributes to quiescence^7^.

Importantly, HSCs are able to adapt rapidly to increased demands triggered by infections, inflammation, chemotherapeutic agents, or ionizing radiation with increased cell cycling and the production of hematopoietic progenitors^8^. This demand-adapted hematopoiesis is activated by immune effector cytokines such as interferon-α, interferon-γ, and tumor necrosis factor-α (TNF-α) and Toll-like receptors (TLRs) that sense conserved microbial products^9,10^.

To ensure lifelong production of all hematopoietic lineages, not only the cell division of HSC but also the cell division fate, for example, differentiation, has to be tightly controlled^11^. Asymmetric cell division, leading to a differentiated and a self-replicable daughter cell, assures a constant stem cell pool and a progressive increase of differentiated cells. Symmetric division leads to two identical daughter cells and the expansion of the stem cell pool or, in rare situations, to two identical more differentiated cells^12^.

Leukemic stem cells (LSCs) are responsible for leukemia initiation and propagation^13^. They share several characteristics with HSCs, including quiescence, self-renewal, and the capacity to undergo symmetric and asymmetric cell division. Importantly, most of the molecular pathways regulating HSCs also control LSCs^14,15^ In addition, we and others showed that the TNF receptor (TNFR) family member CD27 regulates self-renewal and differentiation of HSCs and LSCs^16–19^.

The lymphotoxin-β receptor (LTβR), another member of the TNFR family, is expressed on epithelial, stromal, and myeloid cells but not on lymphocytes. Lymphotoxins are cytokines that guide the interaction of immune cells, mainly lymphocytes, with the surrounding stromal cells^20^. Lymphotoxin-α (LTα) and LTβ as heterotrimers (LTα1β2) and LIGHT are known ligands for LTβR^21^. LTβR signaling is crucially involved in the development and organization of lymphoid tissue^21^. Therefore, secondary lymphoid tissue is absent in *Lta*, *Ltb*, and *Ltbr*-knockout mice but, interestingly, not in *Light*-knockout mice^22,23^. Ligation of LTβR leads to TNFR-associated factor 2 (TRAF2)-NIK-mediated activation of the classical and alternative nuclear factor-κB (NF-κB) pathway and the transcription of genes involved in inflammation and development^24^. Disruption of LTβR signaling results in impaired protection to viral and bacterial infections^25,26^. In addition, cancer cells may express LTβR and agonistic antibodies to LTβR trigger cancer cell death and suppress tumor growth in vivo^27^.

In this work, we document that LIGHT expressed by HSCs induces quiescence and self-renewal by cell-autonomous ligation of LTβR. Importantly, LIGHT expression is upregulated in HSCs in response to increased demands of progenitors and differentiated blood cells. LIGHT/LTβR signaling reduces cell cycling of HSCs, promotes symmetric over asymmetric cell division, and, thereby, maintains the pool of primitive HSCs. Although the maintenance of primitive HSC is crucial to secure hematopoiesis long-term, the accumulation of primitive LSCs and the lack of differentiation promote disease progression in leukemia^28^. Indeed, LIGHT/LTβR signaling maintains and expands the pool of LSCs in a murine model of chronic myeloid leukemia (CML) and promotes disease progression. Similarly, LIGHT/LTβR signaling induces stemness in human CML stem/progenitor cells. Importantly, LIGHT is upregulated in LSCs compared to normal HSCs. This may allow selectively targeting LSCs and inducing differentiation. Collectively, this study identifies the LIGHT/LTβR pathway as a crucial regulator of self-renewal of HSCs and LSCs.

## Results

### LTβR regulates self-renewal of HSCs

**Fig. 1:**
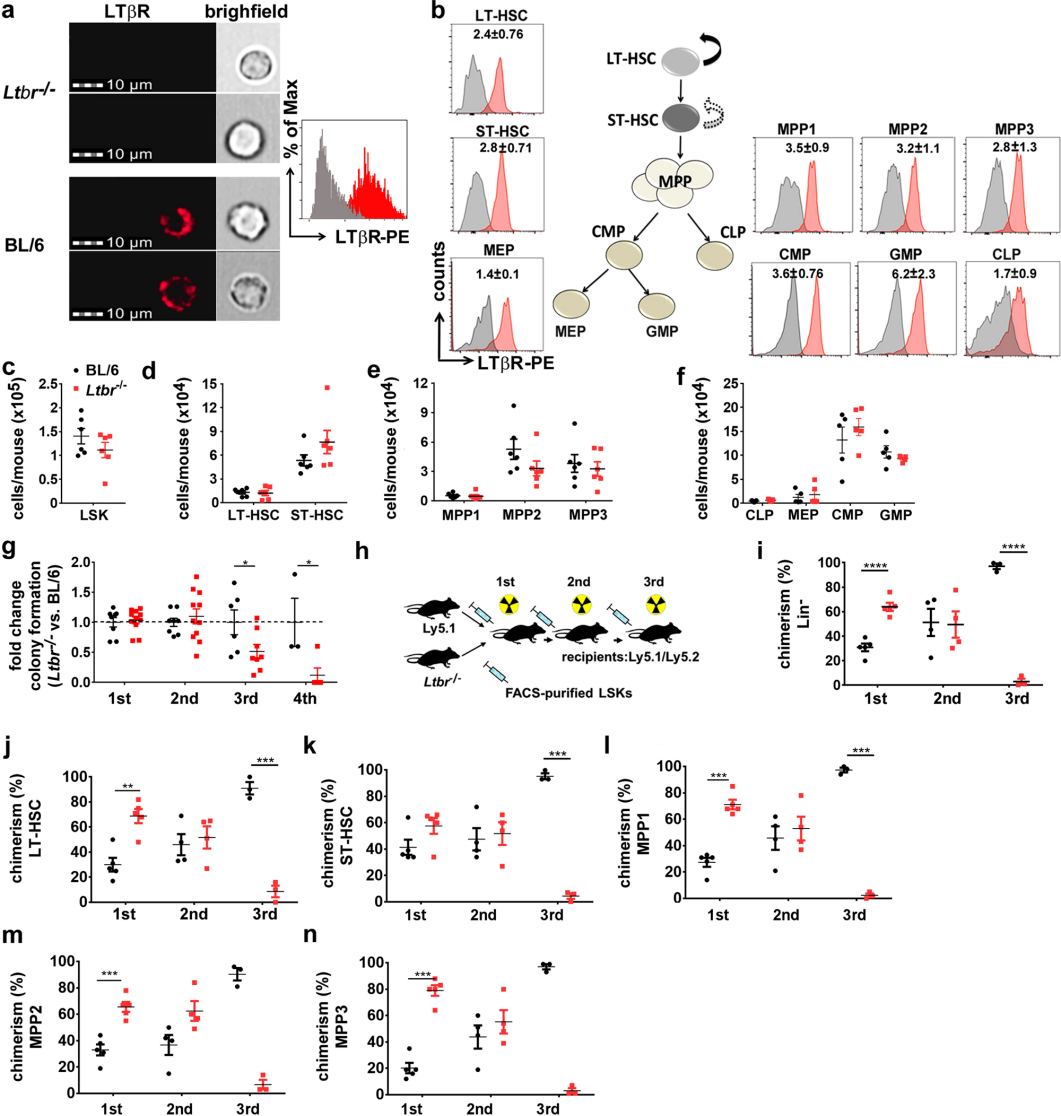
LTβR expressed by LSKs prevents exhaustion of HSCs. **a** Left: LTβR expression (red) on BL/6 and *Ltbr*^−/−^ FACS-purified BM LSKs analyzed by ImageStreamX MkII. Right: ImageStreamX MkII histogram of LTβR expression on *Ltbr*^−/−^ LSKs (gray) vs. BL/6 LSKs (red). **b** Representative FACS histograms of LTβR expression on BM LSK subsets and myeloid progenitor cells. Numbers in plots indicate the ratio MFI stain/isotype (*n* = 5 mice). **c**–**f** Absolute numbers of LSKs (**c**), LT/ST-HSCs (**d**), MPPs (**e**), CLPs and myeloid progenitors (MEP, CMP, GMP) (**f**) in the BM of naive BL/6 (black, *n* = 6) and *Ltbr*^−/−^ (red, *n* = 6) mice. **g** Fold change in CFU of *Ltbr*^−/−^ LSKs relative to BL/6 LSKs in serial re-platings in vitro (1st: *n* = 8 for BL/6 and 11 for *Ltbr*^−/−^; 2nd: *n* = 8 for BL/6 and 11 for *Ltbr*^−/−^; 3rd: *n* = 6 for BL/6 and 8 for *Ltbr*^−/−^; 4th: *n* = 3 for BL/6 and 5 for *Ltbr*^−/−^, one out of two independent experiments). **h** Schematic of mixed BM chimera and serial re-transplantation. **i**–**n** Percentages of Ly5.1 and *Ltbr*^−/−^ Lin^−^ cells (**i**) and LSK subsets (**j**–**n**), *n* = 5 (1st), *n* = 4 (2nd), *n* = 3 (3rd), one out of two independent experiments is shown. Data are shown as mean ± SEM. Statistics: *P < 0.05, **P < 0.01, ***P < 0.001, ****P < 0.0001 (two-tailed *t* test); **g**: 1st *p* = 0.05, 2nd *p* = 0.039; **i**: 1st *p* < 0.0001, 3rd *p* < 0.0001; **j**: 1st *p* = 0.0012, 3rd *p* = 0.0003; **k**: 3rd *p* < 0.0001; **l**: 1st *p* < 0.0001, 3rd *p* < 0.0001; **m**: 1st *p* = 0.0004, 3rd *p* = 0.0001; **n**: 1st *p* < 0.0001, 3rd *p* < 0.0001.

First, we analyzed the expression of LTβR on different murine hematopoietic stem/progenitor cell (HSPC) subsets during homeostasis. ImageStreamX MkII analysis of fluorescence-activated cell sorting (FACS)-purified Lin^−^sca1^+^c-kit^+^ cells (LSKs) from BM revealed a strong surface expression of LTβR (Fig. 1a). In addition, defined LSK subpopulations such as LT-HSCs (CD150^+^CD48^−^CD34^−^CD135^−^), ST-HSCs (CD150^+^CD48^−^CD34^+^CD135^−^), MPPs (MPP1: CD150^+^CD48^+^CD34^+^CD135^−^; MPP2: CD150^−^CD48^+^CD34^+^CD135^−^; MPP3: CD150^−^CD48^+^CD34^+^CD135^+^), as well as common myeloid progenitors (CD34^+^FcγRII/III^−^c-kit^+^sca1^−^), common lymphoid progenitors (CD127^+^c-kit^int^sca1^−^ Thy1.1^−^), granulocyte–macrophage progenitors (CD34^+^FcγRII/III^+^c-kit^+^sca1^−^), and megakaryocyte-erythroid progenitors (CD34^−^FcγRII/III^−^c-kit^+^sca1^−^)^1,29,30^, expressed LTβR as analyzed by FACS (Fig. 1b and Supplementary Fig. 1a). Furthermore, differentiated myeloid cells in the BM and peripheral blood (PB) expressed LTβR, whereas T- and B-lymphocytes only marginally expressed LTβR (Supplementary Fig. 1b).

To analyze the functional relevance of LTβR expressed on hematopoietic cells, we first compared hematopoiesis in naive BL/6 and *Ltbr*^−/−^ mice. BL/6 and *Ltbr*^−/−^ mice had similar numbers of LSKs (LT-HSC, ST-HSC, and MPPs), lymphoid progenitors (common lymphoid progenitors), and myeloid progenitors (granulocyte–macrophage progenitors, common myeloid progenitors, megakaryocyte-erythroid progenitors) in the BM (Fig. 1c–f). In addition, BL/6 and *Ltbr*^−/−^ LSKs had a comparable cell cycle activity and apoptosis rate (Supplementary Fig. 1c, d). Moreover, FACS-purified LSKs from BL/6 and *Ltbr*^−/−^ mice formed similar numbers of colonies in methylcellulose ex vivo (Fig. 1g). Since not only HSCs but also early progenitor cells form colonies ex vivo in methylcellulose^31,32^, we analyzed the self-renewal capacity of HSCs in serial re-plating experiments^33^. *Ltbr*^−/−^ LSKs lost the capacity to form colonies in methylcellulose after the third and fourth re-plating, indicating that LTβR signaling contributes to the self-renewal of HSCs (Fig. 1g).

Since differences between BL/6 and *Ltbr*^−/−^ HSCs only became apparent in serial re-plating assays in vitro that require cell expansion and differentiation, we determined the function of LTβR on LSKs in vivo in a competitive repopulation experiment (Fig. 1h). We first injected congenic *Ltbr*-proficient (Ly5.1^+^, referred to as Ly5.1) LSKs and LTβR-deficient (Ly5.2^+^, referred to as *Ltbr*^−/−^) LSKs into lethally irradiated Ly5.1/Ly5.2 recipient mice. Importantly, injected LSKs homed to the BM independent of LTβR expression (Supplementary Fig. 1e). However, *Ltbr*^−/−^ LSKs reconstituted recipient mice more efficiently than control LSKs, resulting in >60% *Ltbr*^−/−^ Lin^−^ cells in the BM 16 weeks post transplantation (Fig. 1i and Supplementary Fig. 1f). This was also reflected by a significant higher fraction of all HSPC subpopulations (Fig. 1j–n and Supplementary Fig. 1g–j) in the BM and higher numbers of *Ltbr*^−/−^ leukocytes in PB (Supplementary Fig. 1k).

To study the self-renewal capacity of HSCs, we performed serial re-transplantations with equal numbers of FACS-purified *Ltbr*^−/−^ and Ly5.1 LSKs. This resulted in a comparable reconstitution of HSPCs in the second transplantation (Fig. 1i–n and Supplementary Fig. 1f–j) and similar numbers of LTβR-competent and LTβR-deficient leukocytes in blood (Supplementary Fig. 1l). Importantly, *Ltbr*^−/−^ LSKs lost the capacity to reconstitute hematopoiesis in the third transplantation, resulting in only ~3% *Ltbr*^−/−^ HSPCs in BM and differentiated leukocytes in blood (Fig. 1i–n and Supplementary Fig. 1f–j, m). Taken together, these data indicate that LTβR signaling crucially contributes to self-renewal of HSCs.

### Cell-autonomous expression of LIGHT induces LTβR signaling and self-renewal of HSCs

**Fig. 2:**
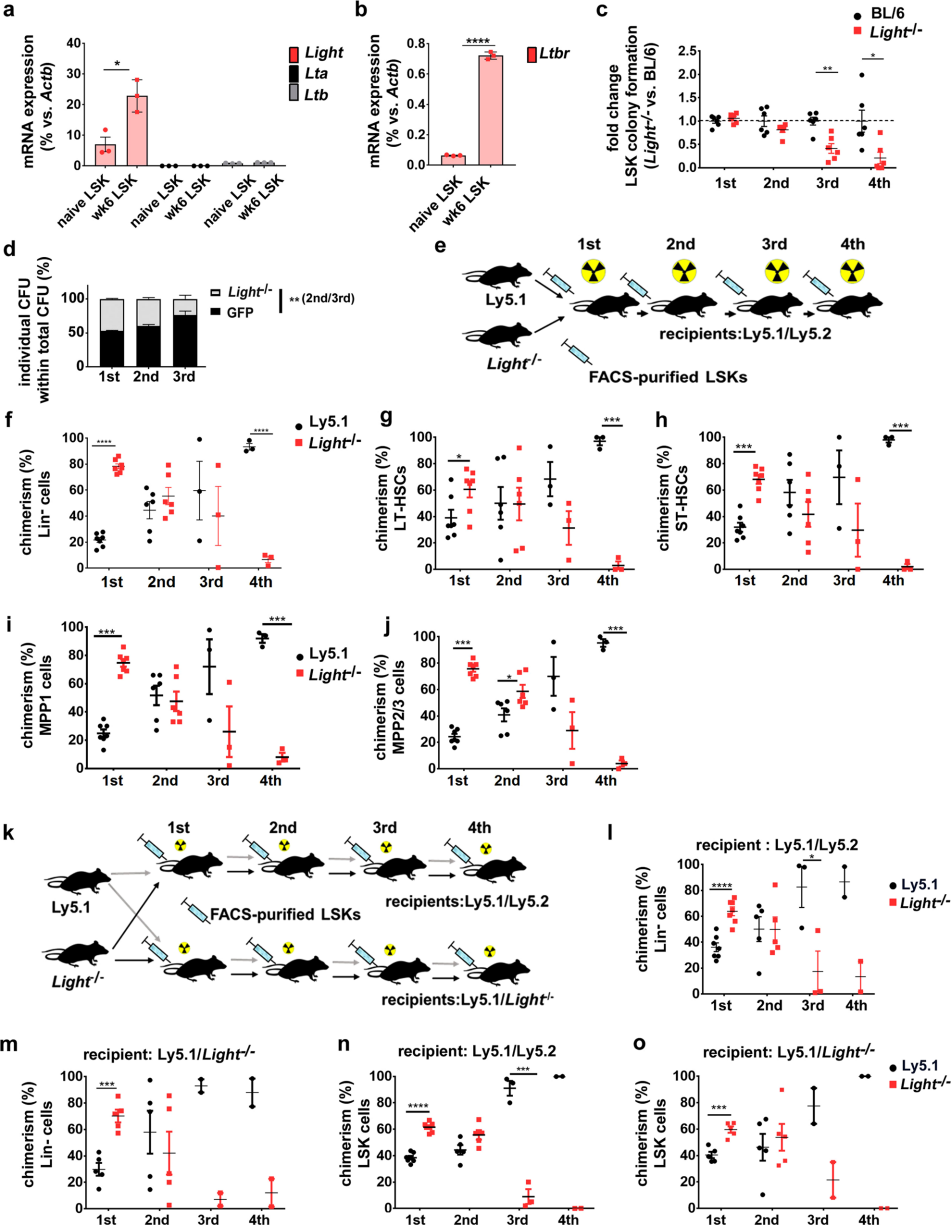
LIGHT expressed by LSKs prevents exhaustion of HSCs. **a** Relative mRNA expression of *Light* (red), *Lta* (black), and *Ltb* (gray) by FACS-purified LSKs from naive mice (*n* = 3) and 1st chimeras 6 weeks post transplantation (*n* = 4 mice, two were pooled for the analysis). **b** Relative mRNA expression of *Ltbr* by FACS-purified LSKs from naive mice (*n* = 3 mice) and 1st chimeras 6 weeks after transplantation (*n* = 4 mice, two were pooled for the analysis). **c** Fold change in colony formation of *Light*^−/−^ BM LSKs (red) relative to BL/6 BM LSKs (black) in serial re-platings (*n* = 6). One out of two independent experiments is shown. **d** Percentage of CFU from *Light*^−/−^ (gray) or BL/6- GFP LSKs (black) in mixed colony-forming assays. The frequency of GFP^+^ (BL/6) vs. GFP^−^ (*Light*^−/−^) colonies is shown. Data are pooled from two independent experiments and presented as mean ± SEM. **e** Experimental scheme for serial transplantations of *Light*^−/−^ Ly5.1 mixed BM chimeras. **f**–**j** Chimerism (red: *Light*^−/−^; black: Ly5.1) in percentages of donor Lin^−^ cells (**f**), LT-HSCs (**g**), ST-HSCs (**h**), MPP1 (**i**), MPP2/3 (Lin^−^c-kit^+^sca1^+^CD150^−^CD48^+^) (**j**). Data are shown as mean ± SEM of *n* = 7 (1st), *n* = 6 (2nd), *n* = 3 (3rd, 4th) mice. **k** Experimental scheme of the serial transplantation of Ly5.1 and *Light*^−/−^ LSKs (1:1) into lethally irradiated Ly5.1/Ly5.2 and congenic Ly5.1/*Light*^−/−^ recipients. **l**–**o** Percentages of donor Lin^−^ cells in Ly5.1/Ly5.2 (**l**) and Ly5.1/*Light*^−/−^ recipients (**m**) and LSKs in Ly5.1/Ly5.2 (**n**), and Ly5.1/*Light*^−/−^ recipients (**o**). Data are shown as mean ± SEM, Ly5.1/Ly5.2 recipients: *n* = 7 (1st), *n* = 5 (2nd), *n* = 3 (3rd); *n* = 3 (4th) Ly5.1/*Light*^−/−^ recipients: *n* = 5 (1st), *n* = 5 (2nd), *n* = 3 (3rd), *n* = 3 (4th). One out of two independent experiments is shown, Statistics: *P < 0.05, **P < 0.01, ***P < 0.001, and ****P < 0.0001 (two-tailed *t* test), **a**: p = 0.005; **b**: p < 0.0001; **c**: p = 0.0014; **d**: p = 0.014, **f**: 1^st^ p < 0.0001, 4th *p* < 0.0001; **g**: 1st *p* = 0.0289, 4th *p* < 0.0001; **h**: 1st *p* < 0.0001, 4th *p* < 0.0001; **i**: 1st *p* < 0.0001, 4th *p* < 0.0001; **j**: *p* < 0.0001, 4th *p* < 0.0001; **l**: 1st *p* < 0.0001, 4th *p* = 0.043; **m**: 1st *p* = 0.0004, 4th *p* < 0.0001; **n**: 1st *p* < 0.0001, 3rd *p* = 0.0005; **o**: *p* = 0.0005. .

LTβR signaling can be induced by ligation with LTα1β2 heterotrimers and LIGHT^34^. Therefore, we analyzed messenger RNA (mRNA) expression of these ligands in naive Ly5.1 LSKs and BM niche cells, such as osteoblasts, endothelial cells (ECs), and mesenchymal stromal cells (MSCs). In addition, FACS-purified Ly5.1 LSKs from chimeras 6 weeks post transplantation were analyzed (Fig. 2a and Supplementary Fig. 2a). *Lta* and *Ltb* mRNA was expressed at very low levels in LSKs from both naive and chimeric mice. However, the membranous form of *Light* was expressed in naive LSKs and its expression was increased ~2.5-fold in Ly5.1 LSKs isolated from chimeric mice (Fig. 2a). By contrast, LTβR ligands were only expressed at low levels in BM niche cells, with the exception of osteoblasts that showed higher *Light* expression (Supplementary Fig. 2a). Interestingly, *Ltbr* mRNA expression in LSKs from chimeric mice 6 weeks post transplantation was increased ~12-fold when compared to naive LSKs (Fig. 2b).

The high expression of LIGHT on LSKs prompted us to analyze its role in the maintenance and regulation of HSPCs. Similar to our results in *Ltbr*^−/−^ mice, the HSPC composition in the BM of naive BL/6 and *Light*^−/−^ mice was comparable (Supplementary Fig. 2b–d). In addition, no differences were found in cell cycle activity and apoptosis rate of LSKs in the absence of *Light* (Supplementary Fig. 2e, f). To further elucidate whether LIGHT-induced LTβR signaling regulates LSK colony formation capacity in vitro, we performed a serial re-plating experiment. In line with our findings obtained with *Ltbr*^−/−^ LSKs, colony formation capacities of *Light*^−/−^ LSKs were gradually lost in serial re-plating experiments in vitro (Fig. 2c).

These data suggest that LIGHT expressed by LSKs triggers LTβR signaling and regulates colony formation in vitro. LTβR expressed on LSKs may bind to LIGHT in *trans* (in which the ligand is expressed on another cell) or in *cis* (the ligand is expressed by the same cell as the receptor). To distinguish between *cis-* and *trans-*signaling, we performed mixed colony assays with FACS-purified green fluorescent protein (GFP)-expressing LIGHT-proficient LSKs and LIGHT-deficient LSKs. LIGHT provided by surrounding LSKs did not rescue the colony-forming capacity of LIGHT-deficient LSKs in serial re-plating experiments (Fig. 2d). This indicates that cell-autonomous LIGHT/LTβR signaling maintains HSC self-renewal capacity.

To study the self-renewal capacity of *Light*^−/−^ LSKs in vivo, we performed competitive serial repopulation experiments (Fig. 2e). *Light*^−/−^ LSKs reconstituted primary recipient mice more efficiently, resulting in a chimerism of ~70% of Lin^−^ BM cells and HSC subsets, as well as higher numbers of leukocytes in blood (Fig. 2f–j and Supplementary Fig. 2g–j). However, *Light*^−/−^ LSKs subsequently lost the capacity to reconstitute hematopoiesis in serial transplantations, resulting in <10% *Light*^−/−^ HSPCs and differentiated leukocytes in the fourth transplantation (Fig. 2f–j and Supplementary Fig. 2g–k). Similarly to the in vitro coculture experiments with LIGHT-proficient and LIGHT-deficient LSKs, LIGHT expressed by surrounding hematopoietic cells did not rescue the defect in self-renewal of LIGHT-deficient LSKs in vivo.

These experiments suggest that cell-autonomous (*cis*) LIGHT/LTβR signaling maintains LSK self-renewal. However, *Light* mRNA was also expressed on osteoblasts (Supplementary Fig. 2a). Therefore, we analyzed whether LIGHT-expressing cells of the BM microenvironment contribute to LTβR signaling in HSCs by transplanting Ly5.1 or *Light*^−/−^ (Ly5.2^+^) LSKs into Ly5.1/Ly5.2 or Ly5.1/*Light*^−/−^ recipients (Fig. 2k). This experiment revealed that LIGHT deficiency on host cells did not alter the reconstitution potential and self-renewal capacity of Ly5.1 HSPCs in serial re-transplantation experiments in vivo. By contrast, *Light*^−/−^ LSKs reconstituted hematopoiesis more efficiently in first transplantation and then gradually lost the capacity to reconstitute hematopoiesis in second, third, and fourth transplantations, independently of the expression of LIGHT in recipient mice (Fig. 2l–o and Supplementary Fig. 2l–o). This indicates that LIGHT ligates LTβR in a cell-autonomous fashion in vivo leading to LT-HSC self-renewal.

### Loss of LTβR increases cell cycle-related gene expression while reducing the expression of genes associated with stemness

**Fig. 3:**
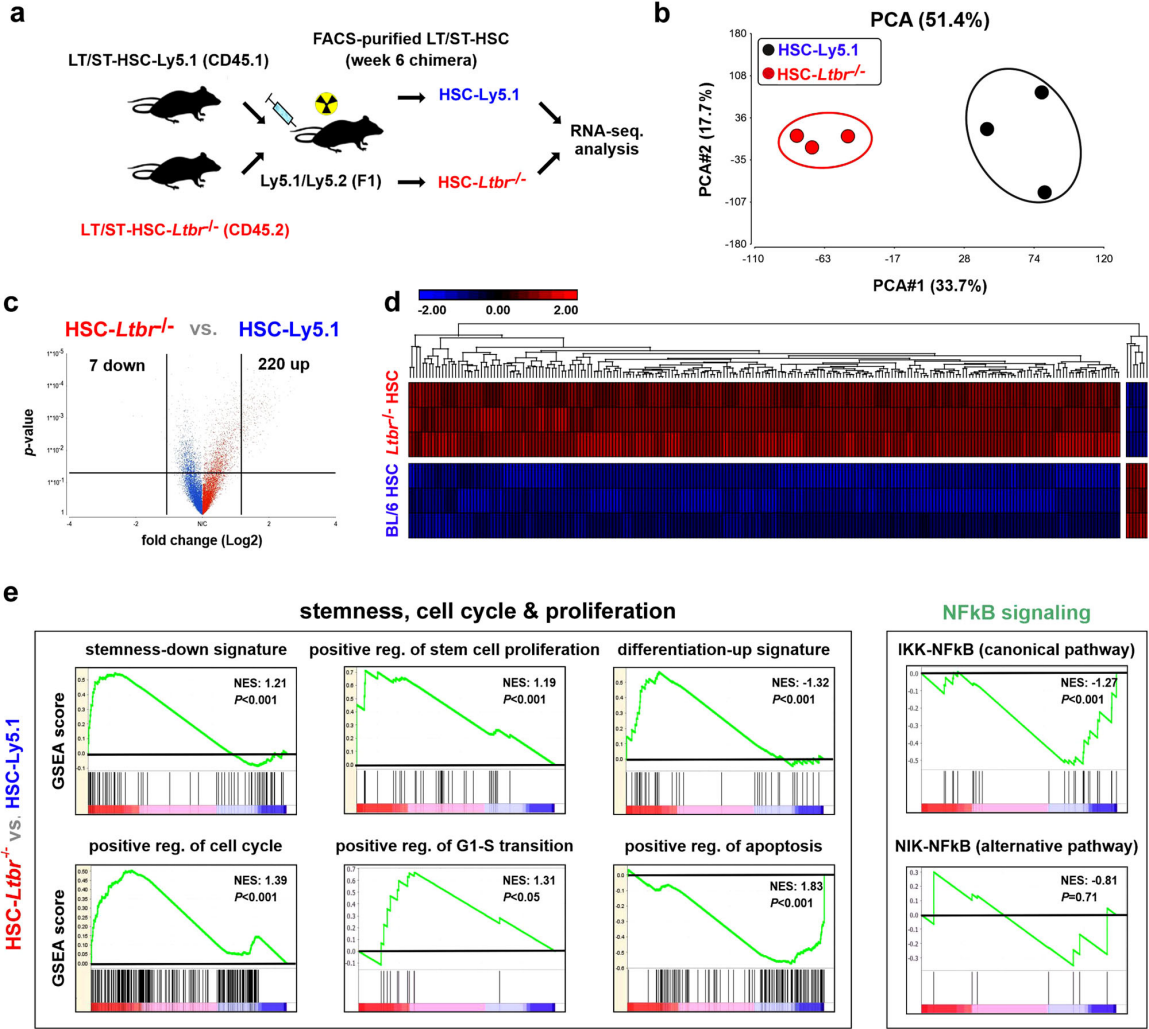
Loss of LTβR leads to a higher expression of genes related to cell cycling and survival while reducing the expression of genes defining stemness. **a** Experimental setting. FACS-purified *Ltbr*^−/−^ and Ly5.1 LT/ST-HSCS from chimeras 6 weeks after primary transplantation (*n* = 6, pooled to three biological replicates) were used for RNA-seq analysis. **b** PCA with samples plotted using the 1st two principal components. **c** Volcano plot of differentially regulated genes. **d** Hierarchical clustering. **e** Gene set enrichment analysis (GSEA, pathcards.genecards.org) .

In order to study the molecular mechanisms of LTβR-mediated HSC regulation, we performed a transcriptomic analysis of Ly5.1 and *Ltbr*^−/−^ LT/ST-HSCs from chimeric mice using RNA-sequencing (RNA-seq) analysis (Fig. 3a). Ly5.1 or *Ltbr*^−/−^ LT/ST-HSCs from different chimeras had similar gene expression profiles and therefore clustered together in a principal component analysis (PCA) (Fig. 3b). Two hundred and twenty-seven genes were differentially expressed between Ly5.1 and *Ltbr*^−/−^ LT/ST-HSCs 6 weeks post transplantation (Fig. 3c and Supplementary data 1). Gene set enrichment analysis (GSEA) revealed a downregulation of stemness- and apoptosis-related gene signatures, but an upregulation of stem cell proliferation, differentiation and cell cycling genes in *Ltbr*^−/−^ vs. control LT/ST-HSCs (Fig. 3e). Gene expression analysis by quantitative reverse transcription PCR revealed an upregulation of genes involved in the cell cycle regulation and differentiation, such as *Myc*, *Ccnd1*, *Cdk4*, and *Numb* in the absence of LTβR signaling. By contrast, stemness-related genes, such as those encoding for RNA-binding protein Musashi 2 (*Msi2*), Kit, and growth factor independent 1 transcriptional repressor (*Gfi1*), were significantly downregulated (Supplementary Fig. 3a).

LIGHT-induced LTβR signaling promotes apoptosis^35^. Similarly, *Ltbr*^−/−^ LT/ST-HSCs and LSKs expressed proapoptotic genes at lower levels and antiapoptotic genes at higher levels compared to Ly5.1 controls (Supplementary Fig. 3a).

In silico network and canonical pathway analysis from LSKs confirmed that LTβR directly and indirectly regulates cell cycle, proliferation, apoptosis, and stemness-related pathways (e.g. Wnt, NF-κB, regulators of HSC activity, and hematopoiesis) (Supplementary Fig. 3b). Interestingly, GSEA of *Ltbr*^−/−^ LT/ST-HSCs for signatures of both NF-κB pathways revealed significant alteration in the canonical NF-κB pathway but not in the signature of the noncanonical pathway (Fig. 3e). Correspondingly, *Traf2* mRNA expression was lower in *Ltbr*^−/−^ vs. Ly5.1 LSKs (Supplementary Fig. 4a, b).

Many of the TNFR ligands including LIGHT have been shown to provide reverse signaling^36^. LIGHT reverse signaling leads to the activation of MAPK/ERK and PI3K-AKT pathways^37^. GSEA revealed no differences in the signatures for MAPK, MAPK/ERK, or PI3K-AKT signaling (Supplementary Fig. 4c). Collectively, our data suggest that LTβR regulates HSC stemness in stress-induced hematopoiesis through activation of the canonical NF-κB pathway.

### LTβR regulates HSC activity and promotes symmetric cell division

**Fig. 4:**
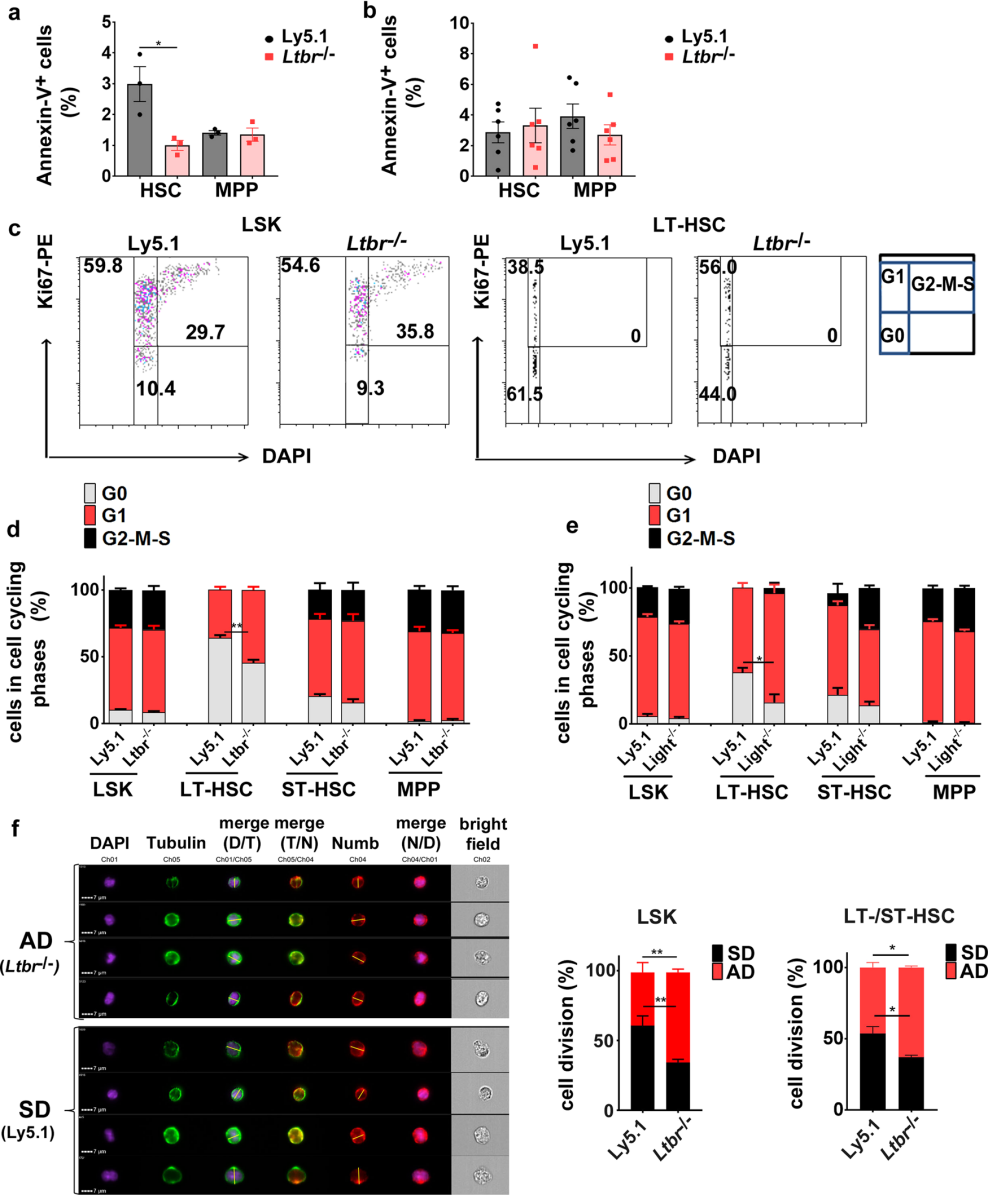
*Ltbr* deficiency induces proliferation and differentiation of HSCs. **a**, **b** Percentages of Annexin-V^+^ HSCs and MPPs 6 (**a**) and 12 (**b**) weeks after 1st transplantation, black: Ly5.1, red: *Ltbr*^−/−^. One out of two independent experiments is shown, for **a**: *n* = 3, for **b**: *n* = 6 mice **c** Representative dot plot of the cell cycle analysis as determined by Ki67 and DAPI staining of Ly5.1 and *Ltbr*^−/−^ BM LSKs and LT-HSCs 12 weeks after primary transplantation. Numbers in plots are shown as percentages of cells in each cell cycle phase, G0 in gray, G1 in red, and G2–M–S in black. **d** Frequency of Ly5.1 and *Ltbr*^−/−^ LSKs, LT-HSCs, ST-HSCs and total MPPs in different cell cycle phases 12 weeks post transplantation. Data are representative for two independent experiments, *n* = 3. **e** Frequency of Ly5.1 and *Light*^−/−^ LSKs, LT-HSCs, ST-HSCs, and total MPPs (CD48^+^) in different cell cycle status 12 weeks post transplantation. LSKs were isolated from two to three chimeric mice and pooled for the analysis. Pooled data from two independent experiments are shown. **f** Representative picture of Numb distribution in dividing Ly5.1 and *Ltbr*^−/−^ FACS-purified LT/ST-HSCs 10–12 weeks after transplantation. DAPI in violet, α-tubulin in green, and Numb in red. Plane cell division (yellow line) was assigned based on α-tubulin and the cleavage furrow. Left: Quantification of Ly5.1 (92 cells from *n* = 3, pooled for the analysis) and *Ltbr*^−/−^ LSKs (135 cells from *n* = 4, pooled for the analysis) in SD (black) or AD (red). Right: Quantification of Ly5.1 (45 cells from *n* = 9, pooled for analysis) and *Ltbr*^−/−^ LT/ST-HSCs (28 cells from *n* = 9, pooled for analysis) in symmetric cell division (SD) or asymmetric cell division (AD). Cells were analyzed by ImageStreamX MkII. Nuclei were stained with DAPI. Data are shown as mean ± SEM. Statistics: *P < 0.05, **P < 0.01 (two-tailed *t* test), **a**: *p* = 0.028; **f** (AD, SD right panel): *p* = 0.033.

We next sought to functionally analyze the mechanisms by which LTβR signaling regulates HSC function. To this end, we analyzed cell viability, cell cycle activity, and cell division of Ly5.1 and *Ltbr*^−/−^ HSCs in chimeric mice. Fewer *Ltbr*^−/−^ HSCs but not MPPs underwent apoptosis when compared to controls 6 weeks after transplantation (Fig. 4a). However, this difference disappeared 12 weeks after transplantation (Fig. 4b).

The reconstitution of hematopoiesis in secondary recipient mice requires that LT-HSCs enter the active cell cycle and expand in numbers by approximately a factor of 10 (refs ^38,39^). To examine cell cycle activity, we FACS-purified Ly5.1 and *Ltbr*^−/−^ LSKs and stained the LSK subsets with Ki67 and 4′,6-diamidino-2-phenylindole (DAPI). LTβR deficiency did not affect cell cycle activity in total LSKs. However, analysis of LSK subpopulations revealed that quiescent (G0) LT-HSCs but not ST-HSCs and MPPs were reduced in the absence of LTβR signaling (Fig. 4c, d). This indicates that LTβR signaling supports quiescence in the most primitive HSC subset, the LT-HSCs. Similarly, *Light*^−/−^ LT-HSCs showed reduced quiescence in a similar experimental setup (Fig. 4e). Taken together, these experiments indicate that LIGHT/LTβR signaling maintains quiescence in primitive HSCs.

Re-entry of HSCs into cell cycle comprises the control between asymmetric and symmetric division that governs differentiation and maintenance of the stem cell pool^6,12^. To assess whether LTβR signaling regulates the cell fate decision of HSPCs, we quantified asymmetric and symmetric cell divisions of Ly5.1 and *Ltbr*^−/−^ LSKs by analyzing the cell fate determinant Numb, a protein known to be distributed either symmetrically or asymmetrically in daughter cells during the division of stem/progenitor cells. A higher Numb expression is associated with differentiation^2,40,41^. The expression of Numb showed a trend towards higher levels in *Ltbr*^−/−^ HSCs compared to Ly5.1 HSCs (Supplementary Fig. 5). The absence of LTβR signaling in FACS-purified LSKs and LT/ST-HSCs promoted asymmetric over symmetric cell division (Fig. 4f). Symmetric cell division can theoretically lead to two stem cells (self-renewal) or to two differentiated daughter cells (commitment)^28^. Overall, this indicates that LIGHT/LTβR signaling in primitive HSCs maintains self-renewal by regulating cell proliferation and asymmetric vs. symmetric cell division.

### LTβR regulates HSC function after genotoxic stress

**Fig. 5:**
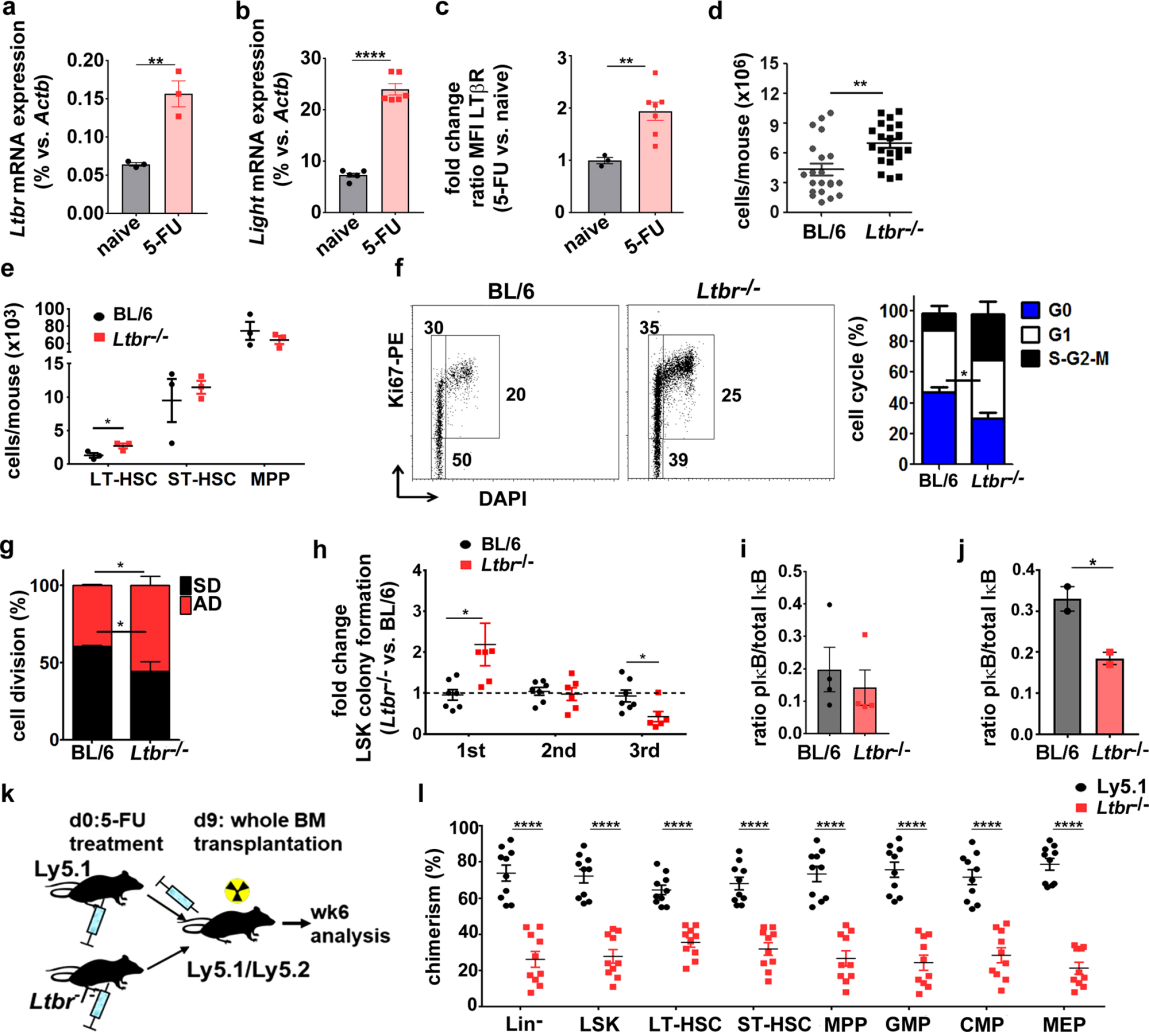
Treatment of *Ltbr*^−/−^ mice with 5-FU leads to increased HSC cell cycle activity and reduced long-term repopulation. **a**, **b** Quantitative RT-PCR analysis of *Ltbr* (**a**) and *Light* (**b**) in BM LSKs from naive (black) and 5-FU-treated (red) BL/6 mice 8 days after treatment (for *Ltbr*: *n* = 3, for *Light*: *n* = 6). **c** Fold change of LTβR MFI on HSCs from 5-FU-treated BL/6 (red, *n* = 7) vs. naive BL/6 mice (black, *n* = 3). **d** Total BM cell numbers of BL/6 (gray) and *Ltbr*^−/−^ mice (black) 8 days post 5-FU injection (BL/6 *n* = 20, *Ltbr*^−/−^ *n* = 21). **e** Total numbers of BM LSK subpopulations in 5-FU-treated BL/6 (black) and *Ltbr*^−/−^ mice (red), *n* = 3. **f** Left: representative FACS profiles of cell cycle analysis of BM LSKs. Right: Percentage of BL/6 and *Ltbr*^−/−^ LSKs in each cell cycle phase, blue: G0, white: G1, black: S–G2–M. Data represent one out of two independent experiments; LSKs were pooled for analysis from BL/6 *n* = 3 and *Ltbr*^−/−^ *n* = 5 mice. **g** Percentages of FACS-purified LSKs from 5-FU-treated BL/6 and *Ltbr*^−/−^ mice in symmetric (SD, black) or asymmetric division (AD, red). Data represent one out of two independent experiments, LSK numbers *n* = 73 (BL/6) and *n* = 85 (*Ltbr*^−/−^). **h** Fold change of CFU capacity of FACS-purified BL/6 (black) and *Ltbr*^−/−^ (red) LSKs in serial re-plating experiments, pooled data from two independent experiments are shown (BL/6 *n* = 5, *Ltbr*^−/−^ *n* = 7). **i** Ratio of IκBα protein expression and its phosphorylation level of BM LT/ST-HSCs from naive BL/6 (black) and *Ltbr*^−/−^ mice (red). Data were pooled from two independent experiments (BL/6 *n* = 5 mice, *Ltbr*^−/−^ *n* = 4 mice). **j** Ratio of IκBα protein expression and its phosphorylation level of BM LT/ST-HSCs (Lin^−^sca^−^1^+^CD48^−^CD150^+^) from BL/6 and *Ltbr*^−/−^ mice 8 days post treatment with 5-FU, *n* = 2 mice were pooled for each data point. One out of two independent experiments is shown. **k** Experimental setup of BM reconstitution after 5-FU treatment. **l** Percentages of Ly5.1 (black) and *Ltbr*^−/−^ BM donor cells (red) of recipients 6 weeks post transplantation, one out of two independent experiments (*n* = 10). Unless otherwise stated, data are presented as mean ± SEM, *P < 0.05, **P < 0.01, ****P < 0.0001 (two-tailed *t* test). **a**: *p* = 0.0058; **b**: *p* < 0.0001; **c**: *p* = 0.0092; **d**: 0.0039; **e**: *p* = 0.030; **f**: 0.025; **g**: *p* = 0.042; **h**: 1st *p* = 0.031, 3rd *p* = 0.025; **i**: *p* = 0.55; **j**: *p* = 0.047; **l**: *p* < 0.0001.

Genotoxic drugs or irradiation lead to damage and eradication of hematopoietic cells and results in the activation of a demand-adapted hematopoiesis in order to replenish the hematopoietic system. To test hematopoietic reconstitution after genotoxic stress, we treated BL/6 and *Ltbr*^−/−^ mice with fluorouracil (5-FU)^42^. Since treatment with 5-FU leads to downregulation of c-kit^43^, LSKs and all HSCs subsets were gated within the Lin^−^sca-1^+^ fraction (Supplementary Fig. 6a). 5-FU treatment resulted in increased *Ltbr* and *Light* mRNA and LTβR protein expression in LSKs (Fig. 5a–c). Interestingly, total BM cellularity was significantly higher in *Ltbr*^−/−^ compared to BL/6 mice 8 days after 5-FU treatment (Fig. 5d). In addition, the number of LT-HSCs was significantly increased in *Ltbr*^−/−^ compared to BL/6 mice, whereas the number of ST-HSCs and MPPs remained unchanged (Fig. 5e). In accordance with the reduced apoptosis rate of *Ltbr*^−/−^ HSCs in chimeric mice (Fig. 4a), significantly fewer *Ltbr*^−/−^ HSCs and CD150^+^/CD48^+^ MPPs stained Annexin-V^+^ compared to controls (Supplementary Fig. 6b). In addition, significantly fewer *Ltbr*^−/−^ LSKs were in the G0 phase of the cell cycle compared to BL/6 LSKs (Fig. 5f). Moreover, *Ltbr* deficiency resulted in an increase in asymmetric over symmetric cell division (Fig.5g). LSKs from *Ltbr*^−/−^ mice initially formed significantly more colonies 8 days after 5-FU treatment. Importantly, HSC and progenitors form colonies in the first plating. However, colony formation was consecutively lost in serial re-platings, suggesting a loss of HSCs with self-renewal capacity (Fig. 5h).

The phosphorylation and degradation of IκBα is a key process resulting in NF-κB activation^44^. IκBα was expressed at higher levels in naive and in 5-FU-treated *Ltbr*^−/−^ LT/ST-HSCs (Supplementary Fig. 6d, f). Importantly, the pIκBα/IκBα ratio was similar in naive *Ltbr*^−/−^ LT/ST-HSCs but significantly reduced after 5-FU treatment when compared to control HSCs (Fig. 5i, j). This indicates that, in response to 5-FU treatment, LTβR signals via the canonical NF-κB pathway.

Since LTα1β2 is an additional ligand for LTβR and LIGHT also ligates herpesvirus entry mediator (HVEM), we analyzed a possible role of additional ligands/receptor in *Light* and *Ltbr* double knockout (KO) mice. *Light*^−/−^/*Ltbr*^−/−^ LSKs had a similar phenotype in colony formation compared to single KO LSKs, suggesting that LIGHT/LTβR acts as a ligand/receptor pair and excluding a major role of other ligands/receptors (Supplementary Fig. 6g, h).

To study the number and function of HSCs after 5-FU treatment in vivo, we transplanted BM cells from 5-FU-treated Ly5.1 and *Ltbr*^−/−^ donors into lethally irradiated Ly5.1/Ly5.2 mice (Fig. 5k). *Ltbr*^−/−^ donor BM cells reconstituted recipient mice less efficiently than control BM cells, resulting in a significantly reduced frequency of Lin^−^ cells and HSPCs (Fig. 5l). These findings suggest that the frequency of HSCs capable of reconstituting recipient mice is significantly lower in 5-FU-treated *Ltbr*^−/−^ mice than in controls. Together, these data indicate that LIGHT/LTβR signaling reduces cell proliferation and asymmetric cell division and thereby maintains the pool of HSCs in response to genotoxic stress.

### LIGHT/LTβR signaling in LSCs promotes CML development

**Fig. 6:**
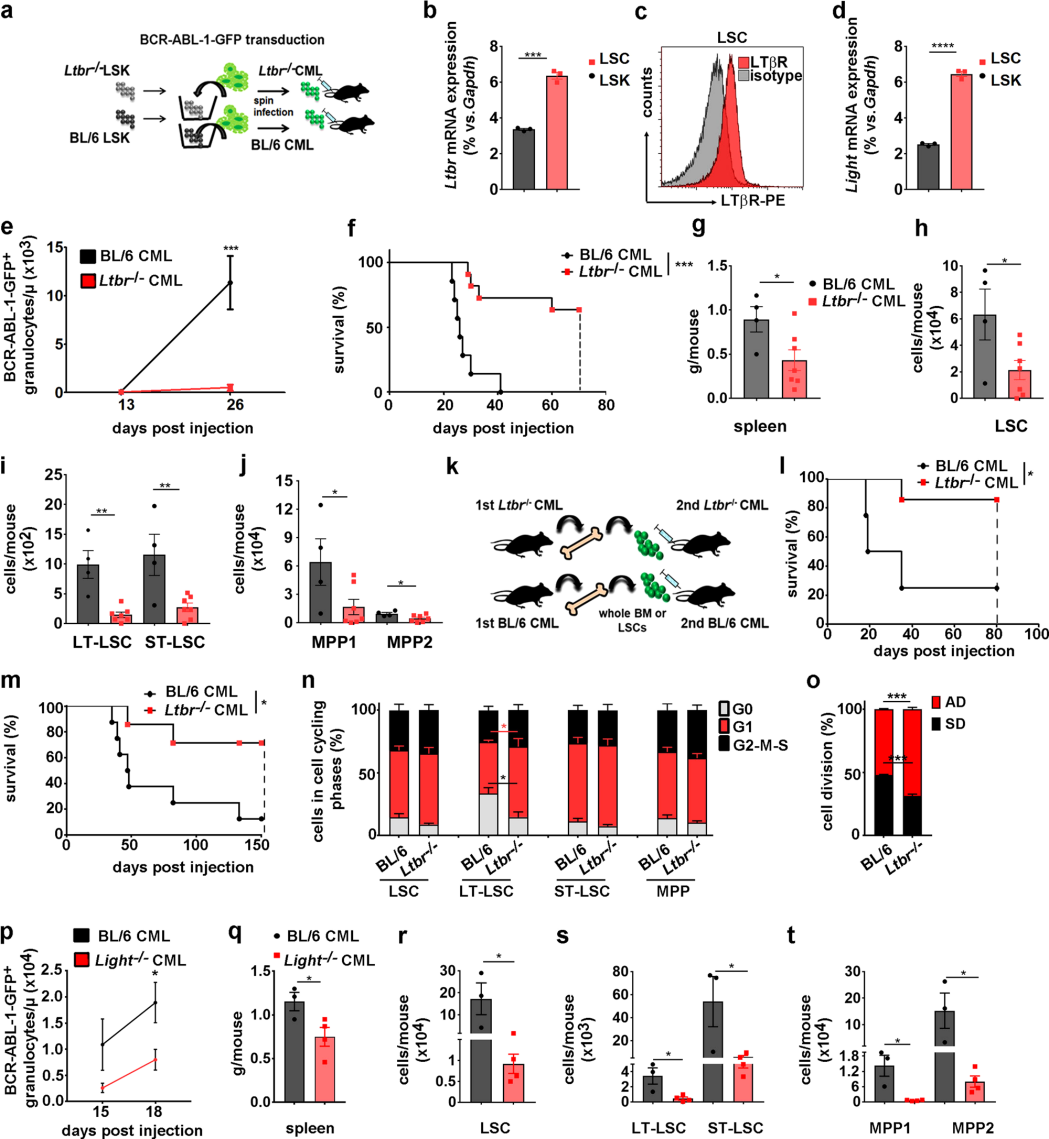
LTβR expression of LSCs promotes CML progression. **a** Schematic for CML induction. **b** *Ltbr*^−/−^ mRNA expression in naive BL/6 LSKs (black) and LSCs (red, Lin^−^GFP^+^c-kit^+^sca1^+^, *n* = 3). **c** LTβR expression on BL/6 LSCs. **d** *Light* mRNA expression of naive BL/6 LSKs (black) and BL/6 LSCs (red, *n* = 3). **e** BCR/ABL1-GFP^+^ granulocytes/μl in blood from BL/6 (black, *n* = 12) and *Ltbr*^−/−^ CML mice (red, *n* = 13). One out of two independent experiments is shown. **f** Kaplan–Meier survival curves of BL/6 (*n* = 7) and *Ltbr*^−/−^ CML mice (*n* = 11, dashed line: predetermined endpoint of the experiment). **g** Spleen weight of BL/6 (black, *n* = 4) and *Ltbr*^−/−^ CML mice (red, *n* = 7). **h**–**j** LSC (**h**), LT-LSC, ST-LSC (**i**), and leukemic MPPs (**j**) cell numbers in BM of BL/6 and *Ltbr*^−/−^ mice. Data are shown as one out of three independent experiments. **k** Schematic of secondary CML transplantation with BM cells or LSCs. **l**, **m** Kaplan–Meier survival curves for whole CML BM cells (**l**, BL/6 *n* = 4, *Ltbr*^−/−^ *n* = 7) and LSCs (**m**, BL/6 *n* = 8, *Ltbr*^−/−^ *n* = 7). **n** Percentage of LT-HSC, ST-HSC, and MPPs from BL/6 and *Ltbr*^−/−^ mice in G0 (gray), G1 (red), and G2–M–S (black). Data are pooled from two independent experiments (*n* = 6). **o** Percentages of BL/6 and *Ltbr*^−/−^ LSCs in symmetric (SD, black) or asymmetric division (AD, red), *n* = 140 cells (BL/6) and *n* = 230 cells (*Ltbr*^−/−^) were examined in two independent experiments. Data are shown as mean ± SEM. **p** BCR/ABL1-GFP^+^ granulocytes/μl in blood from BL/6 (black, *n* = 6) and *Light*^−/−^ CML mice (red, *n* = 8). One out of two independent experiments is shown. **q** Spleen weight of BL/6 (black, *n* = 3) and *Light*^−/−^ CML mice (red, *n* = 4). **r**–**t** LSC (**r**), LT-LSC, ST-LSC (**s**), and leukemic MPPs (**t**) cell numbers in BM of BL/6 (*n* = 3) and *Light*^−/−^ CML mice (*n* = 4). Data are shown as mean ± SEM. Statistics: *P < 0.05, **P < 0.01, ***P < 0.001, and ****P < 0.0001 (two-tailed *t* test: **b**, **d**, **e**, **g**, **h**–**j**, **n**–**p**, **q**–**t**), two-tailed log-rank test (**f**, **l**, **m**), **b**: *p* = 0.0001; **d**: *p* < 0.0001; **e**: *p* = 0.0001; **f**: *p* = 0.0002; **g**: *p* = 0.038; **h**: *p* = 0.036; **i**: LT-LSC *p* = 0.0011; ST-LSC *p* = 0.0095; **j** MPP1 *p* = 0.049, MPP2 *p* = 0.04; **l**: *p* = 0.028; **m**: *p* = 0.0196; **n**: G0 *p* = 0.014, G1 *p* = 0.044; **o**: SD/AD *p* = 0.0003; **p**: *p* = 0.021; **q**: *p* = 0.048; **r**: *p* = 0.043; **s**: LT-LSC *p* = 0.024, ST-LSC *p* = 0.048; **t**: MPP1 *p* = 0.012, MPP2 *p* = 0.048.

So far, our data indicated that LTβR signaling regulates HSC cell cycling and self-renewal. Since self-renewal and regulation of cell fate in LSCs is crucial for the development of the disease, we next studied LTβR signaling in a murine CML model^45^ CML-like disease was induced by injection of BCR-ABL1-GFP-transduced BL/6 or *Ltbr*^−/−^ LSKs into nonirradiated BL/6 recipients (Fig. 6a). In this model, LSCs are characterized as GFP^+^LSKs and LSC subsets are defined in analogy to normal HSC subpopulations^18^ (Supplementary Fig. 7a).

LSCs expressed *Ltbr* mRNA at significantly higher levels than normal LSKs (Fig. 6b). FACS analysis revealed that LTβR was expressed on LSCs and leukemia progenitors with the highest expression on LT-LSCs (Fig. 6c and Supplementary Fig. 7b–d). In addition, LSCs expressed *Light* at higher levels than normal LSKs (Fig. 6d). *Ltbr*^−/−^ CML progressed significantly slower than BL/6 CML with reduced leukemia granulocytes in PB leading to a prolonged survival (Fig. 6e, f). In addition, spleen weights of *Ltbr*^−/−^ CML mice were significantly reduced compared to BL/6 CML mice 19 days post transplantation, indicating a lower leukemia burden (Fig. 6g). Importantly, significantly fewer LSCs (LT- and ST-LSCs) and MPPs were found in the BM of *Ltbr*^−/−^ compared to BL/6 CML mice 19 days after transplantation (Fig. 6h–j).

In order to functionally analyze the role of LTβR signaling in LSCs, we transplanted either whole BM or FACS-purified LSCs from primary BL/6 and *Ltbr*^−/−^ CML mice into nonirradiated BL/6 recipients (Fig. 6k). Recipients of *Ltbr*^−/−^ CML BM (Fig. 6l) or LSCs (Fig. 6m) survived significantly longer than recipients of BL/6 BM or LSCs, respectively. This indicates that *Ltbr*^−/−^ CML harbors fewer LSCs and that the phenotypically characterized *Ltbr*^−/−^ LSCs are functionally impaired.

Lack of LTβR on LSCs increased cell cycle activity with an ~2-fold decrease of LT-LSCs in G0 phase compared to BL/6 LT-LSCs (14.7 ± 10.2 vs. 33.4 ± 11.6). By contrast, cell cycle activity of ST-LSCs or MPPs did not depend on LTβR signaling (Fig. 6n). Moreover, the analysis of the distribution of Numb in dividing LSCs in telophase revealed an increased number of *Ltbr*^−/−^ LSCs in asymmetric over symmetric cell division (Fig. 6o). Moreover, Numb expression is higher in *Ltbr*^−/−^ LSCs compared to BL6 LSCs (Supplementary Fig. 7e).

Similarly, CML induced by *Light*^−/−^ BCR-ABL1-GFP LSKs (*Light*^−/−^ CML) progressed significantly slower than BL/6 CML with reduced numbers of leukemia granulocytes in PB and significantly reduced spleen weights 18 days post transplantation (Fig. 6p, q). In addition, significantly fewer LSCs (LT- and ST-LSCs) and MPPs were found in the BM of *Light*^−/−^ compared to BL/6 CML mice 18 days post transplantation (Fig. 6r–t). *S*upplementation of recombinant LIGHT in the colony-forming assay did not rescue the KO phenotype (Supplementary Fig. 7f). This finding is in agreement with the documented role of cell-autonomous LIGHT/LTβR (*cis*) signaling. These data indicate that LIGHT/LTβR signaling regulates cell division and cell fate in LSCs and promotes disease progression.

### LTβR signaling regulates stemness in human CD34^+^ HSPCs

**Fig. 7:**
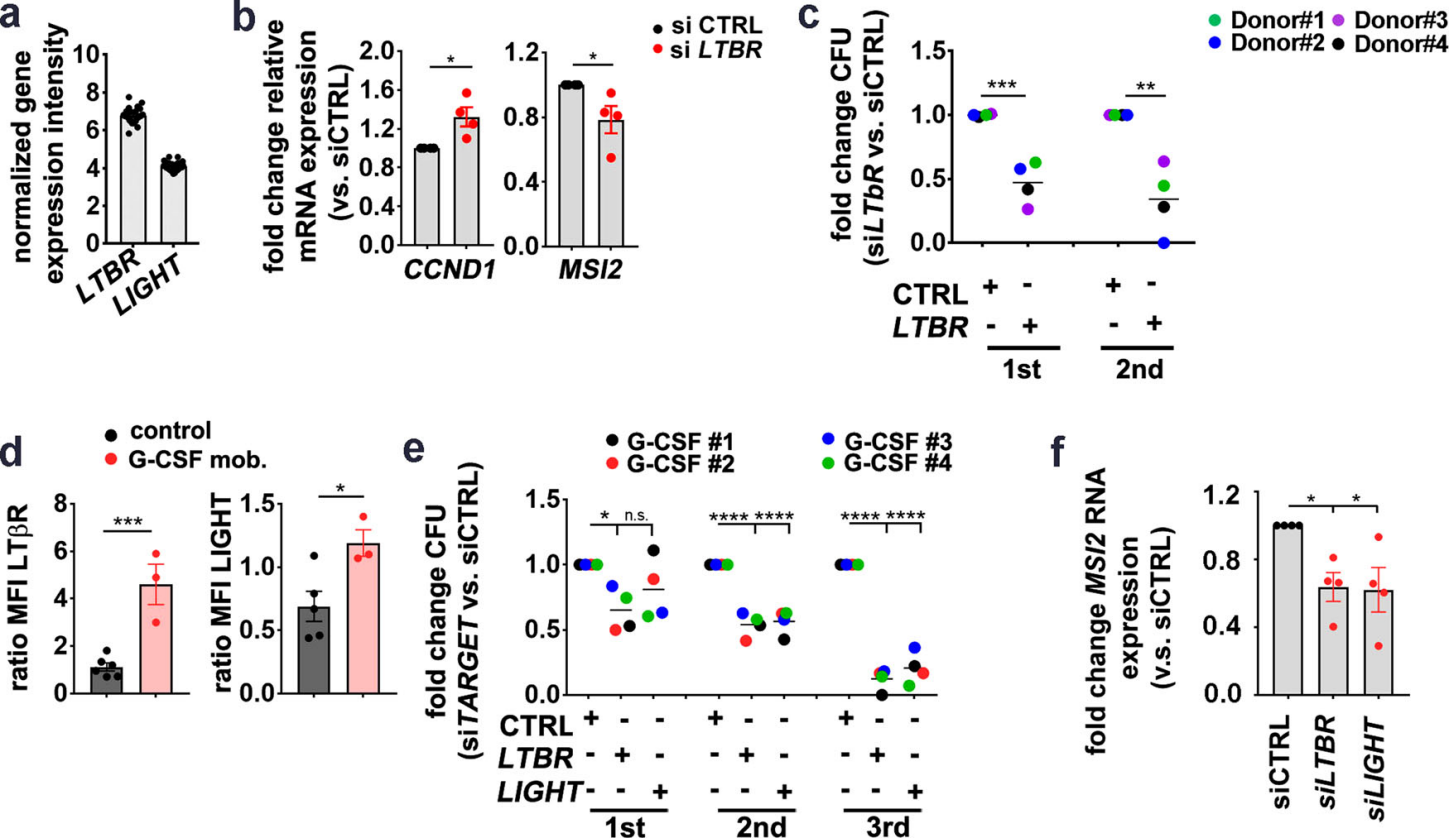
LTβR signaling in human HSPCs. **a** mRNA expression intensity of *LTBR* and *LIGHT* in CD34^+^ cells from 27 healthy donors, analyzed in a publicly available microarray dataset (GSE32719). **b** Fold change of relative mRNA expression of indicated genes in HSPCs (CD45^int^Lin^−^CD34^+^) from control samples, transfected with si*LTBR* (red) relative to HSPCs treated with siCTRL (black), *n* = 4, pooled for analysis from three independent experiments. **c** Fold-change CFU and re-plating of si*LTBR*- or siCTRL-transfected BM HSPCs (*n* = 4, pooled for analysis from two independent experiments, shown as grand mean). **d** MFI (stain/isotype) of LTβR and LIGHT in BM HSPCs from control samples (black, *n* = 6) and G-CSF-mobilized PB HSPCs (red, *n* = 3). Data from five independent experiments were pooled for analysis. **e** Fold-change CFU and re-plating of PB HSPCs from G-CSF-treated patients, transfected with indicated siRNA (*n* = 4). Data are pooled for analysis from two independent experiments, shown as grand mean. **f** Fold change of *MSI2* mRNA in HSPCs from G-CSF patients, transfected with si*LTBR* (red) or si*LIGHT* (red) relative to HSPCs treated with siCTRL (black) (*n* = 4, pooled data for analysis from two independent experiments). Data are presented as mean ± SEM. Statistics: *P < 0.05, **P < 0.01, ***P > 0.001 (two-tailed *t* test: **b**–**d**, and one-way ANOVA: **e**, **f**). **b**: CCND1 *p* = 0.018, MSI2 *p* = 0.043; **c**: 1st *p* = 0.0007, 2nd *p* = 0.0028; **d**: LTβR *p* = 0.0007, LIGHT *p* = 0.030; **e**: 1st *p* = 0.04, 2nd *p* < 0.0001, 3rd *p* < 0.0001; **f**: CTRL vs. si*LTBR* *p* = 0.035, CTRL vs. si*LIGHT* *p* = 0.029. n.s. Not significant.

*LTBR* and *LIGHT* are expressed by human CD34^+^ BM cells on the mRNA level (GEO: GSE32719)^46^ (Fig. 7a). Next, we silenced LTβR expression in FACS-purified BM CD34^+^ HSPCs from untreated staging negative lymphoma patients (control samples) using small interfering RNA (siRNA) (Supplementary Fig. 8a). *LTBR* knockdown significantly increased the expression of genes related to proliferation, such as *CCND1*, but reduced stemness-related gene such as *MSI2* (Fig. 7b). In addition, CD34^+^ BM cells treated with siRNA for 24 h formed significantly fewer colonies in methylcellulose and lost the capacity to form colonies in serial re-platings, indicating that the knockdown of *LTBR* reduced the number of functional HSCs (Fig. 7c).

**Fig. 8:**
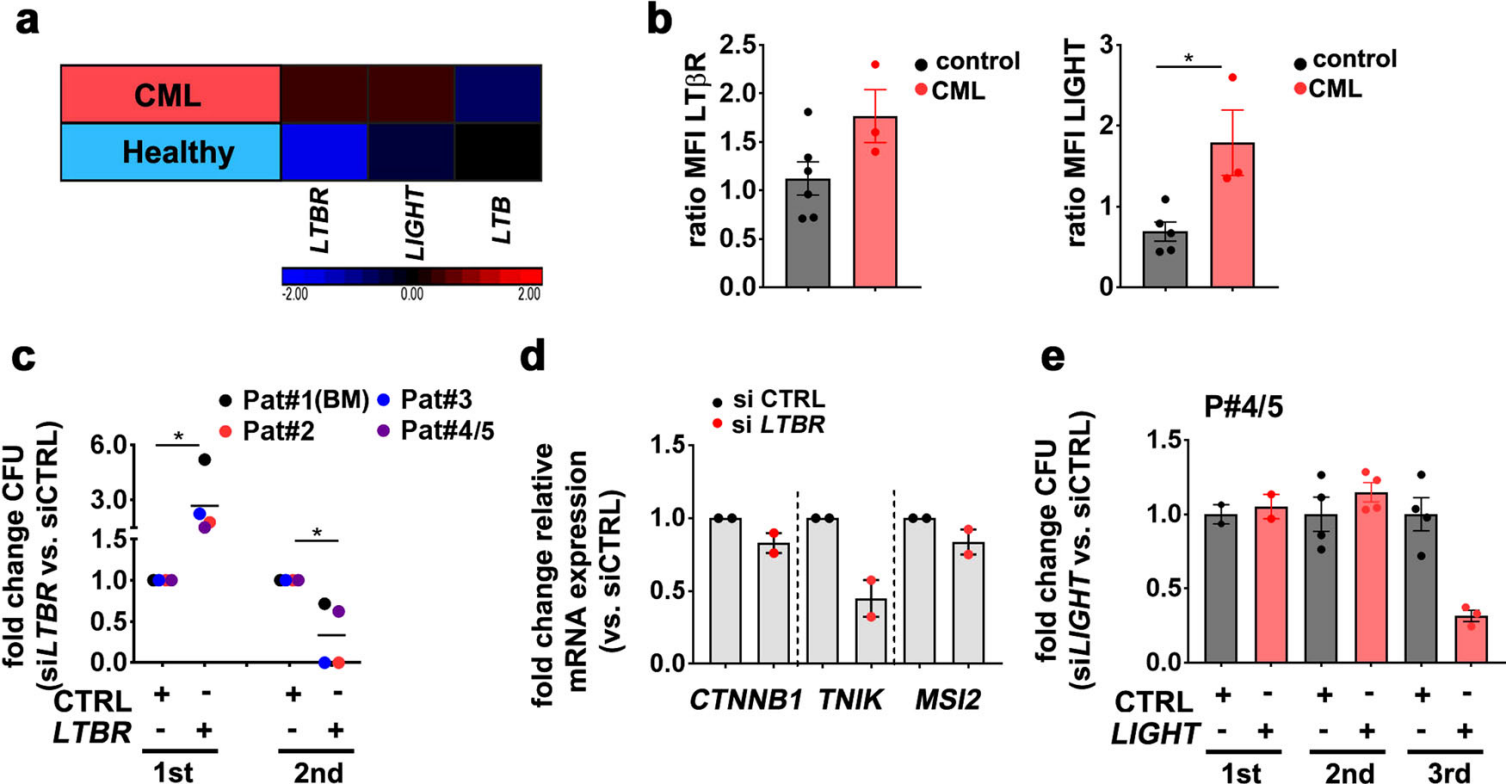
LTβR signaling in CD34^+^ HSPCs from CML patients. **a** Heatmap analysis for *LTBR*, *LIGHT*, and *LTB* gene expression (log 2 fold differences) in PB CD34^+^ Lin^−^ cells from CML patients (red) and healthy donors (blue) of a publicly available dataset (GEO: GSE11675). **b** MFI of LTβR and LIGHT in BM HSPCs (black, *n* = 6) and BM HSPCs from CML patients (red, *n* = 3). Data are pooled for analysis from five independent experiments. **c** Fold change CFU and re-plating of CML HSPCs, transfected with si*LTBR* vs. siCTRL (*n* = 4, one sample (Pat 4/5) was used as pooled cells from two CML patients. Data are pooled for analysis from two independent experiments and shown as grand mean. **d** Fold change of mRNA expression of indicated genes in si*LTBR* (red)-transfected CML HSPCs, relative to siCTRL (black)-treated CML HSPCs (*n* = 4, data are pooled for analysis from two independent experiments. **e** Fold-change CFU and re-platings of si*LIGHT* (red)-transfected CML HSPCs, relative to siCTRL (black). Cells were pooled from *n* = 2 CML patients. Data are presented as mean ± SEM. Statistics: *P < 0.05 (two-tailed *t* test: **b**, **c**). **b**: LTβR, *p* = 0.07, LIGHT, *p* = 0.030; **c**: 1st, *p* = 0.018, 2nd, *p* = 0.029.

To analyze the role of LIGHT/LTβR signaling in activated hematopoiesis, we isolated CD34^+^ HSPCs from PB of patients that have been treated with cyclophosphamide mobilization chemotherapy and granulocyte colony-stimulating factor (G-CSF). Interestingly, LTβR and LIGHT expression were increased in G-CSF-mobilized CD34^+^ HSPCs compared to control BM CD34^+^ HSPCs (Fig. 7d). *LTBR* knockdown in G-CSF-mobilized CD34^+^ HSPCs reduced colony-forming unit (CFU) capacity in the first, second, and third plating in methylcellulose. By contrast, *LIGHT* siRNA treatment resulted in similar numbers of colonies in the first plating, but significantly fewer colonies after re-plating (Fig. 7e). As expected, siRNA treatment did only transiently downregulate LTβR and LIGHT with reconstituted expression after the first plating (Supplementary Fig. 8d). This indicates that transient silencing of LTβR or LIGHT leads to differentiation in the first plating with fewer HSCs capable of self-renewing in the second and third re-plating. *LTBR* and *LIGHT* knockdown significantly reduced the expression of the stemness-related gene *MSI2* in G-CSF-mobilized HSPCs (Fig. 7f). Importantly, the expression of *MSI2* positively correlated with the expression of *LTBR* and *LIGHT* after knockdown (Supplementary Fig. 8e, f). To analyze the expression of *LTBR*, *LTA*, *LTB*, and *LIGHT* in CD34^+^ CML stem/progenitor cells, we took advantage of a public available microarray dataset (GEO: GSE11675)^47^. While *LTB* was similarly expressed in normal and in CML CD34^+^ cells, *LIGHT* and *LTBR* expression was clearly increased in CML samples (Fig. 8a). In accordance, FACS analysis of CD34^+^ HSPCs from CML patients revealed a significantly higher LIGHT expression and a trend to a higher expression of LTβR than in control CD34^+^ HSPCs (Fig. 8b). *LTBR* knockdown in CD34^+^ CML cells resulted in more colonies in the first plating in methylcellulose but fewer colonies in the second re-plating (Fig. 8c and Supplementary Fig. 8g). Importantly, knockdown of *LTBR* reduced the expression of *MSI2*, *CTNNB1*, and *TNIK*, indicating reduced stemness (Fig. 8d).

*LIGHT* knockdown in CML CD34^+^ HSPCs resulted in a reduced CFU capacity after re-plating (Fig. 8e and Supplementary Fig. 8h). Collectively, these data indicate that LIGHT/LTβR signaling contributes to the maintenance and self-renewal of human hematopoietic and CML stem/progenitor cells.

## Discussion

In response to an increased demand of blood cells, HSPCs are activated to enter cell cycling, proliferation, differentiation, and migration^48^. This activation is regulated by paracrine signals from the HSC niche cells including ECs and BM MSCs and from immune cells of the BM microenvironment^6,48^. For example, type I and type II IFNs, G-CSF, and LPS are important activators of the hematopoiesis in response to increased demand^10^. In addition, HSCs sense pathogen-associated molecular patterns during infection mainly via TLRs and respond with proliferation and myeloid differentiation^49^. Danger signals such as TLR signaling have been shown to induce the autocrine production of hematopoietic cytokines in HSPC^50^. However, primitive HSCs cannot be activated to proliferate indefinitely since this will lead to exhaustion of HSCs and loss of LT hematopoiesis^51^. Therefore, signaling pathways that regulate HSCs quiescence and cell fate are crucial for the maintenance of the HSC pool. More than 200 genes have been identified to regulate HSC function^52^. They are mainly involved in the regulation of cell cycling, Pten/Akt, and Wnt pathway^53–55^. In addition, external cues maintain HSC quiescence and self-renewal.

Different TNFRs have been implicated in the regulation of hematopoiesis. TNF-α is a major regulator of demand-adapted hematopoiesis via signaling through the p55 TNFR 1α^56^. CD40L stimulates human cord blood HSPC proliferation and myeloid differentiation^57^. In addition, CD70-expressing immune cells regulate HSPC function and differentiation during infection via CD27 signaling^16,17^. CD27, CD40, and LTβR signal via TRAFs 2 and 5, which activate multiple signaling pathways, including the NF-κB pathway^58–60^. Our GSEA and a conformational analysis of the phosphorylation status of IκBα revealed that LIGHT/LTβR preferentially signals via the canonical NF-κB pathway. Importantly, the NF-κB pathway has been documented before as a central regulator of HSC maintenance and homeostasis^61^.

In addition, CD27 signaling contributes to the maintenance and expansion of LSCs via TRAF2/TNIK signaling and Wnt pathway activation^18^. CD27 and LTβR signaling is mainly regulated by the expression of its respective ligands. The CD27 ligand CD70 is only expressed on lymphocytes and subsets of dendritic cells upon activation^62^. However, permanent CD70 expression on LSCs allows cell-autonomous CD27 signaling and expansion of LSCs^63^. Similarly, LIGHT is expressed mainly on immune cells upon activation^64,65^. Here, we show that HSPCs express LIGHT and that its expression is upregulated upon activation. Interestingly, LTβR is also expressed at higher levels on activated HSCs. Our in vitro coculture experiments and competitive BM transplant models using *Light*^−/−^ and control LSKs indicated that LTβR and LIGHT act in *cis* in HSCs and this interaction is crucial for the regulation of self-renewal. In accordance with our findings in HSCs, soluble LIGHT added to *Light*^−/−^ CML LSCs did not rescue colony formation capacity.

LIGHT interacts with three different receptors (HVEM, LTβR, and decoy receptor 3), two of which are expressed on HSPCs^66^. HVEM signaling in murine HSPCs has been shown to induce myeloid differentiation in response to treatment with LIGHT in vitro and in vivo^66^. Here we show that signaling via LIGHT/LTβR maintained the pool of HSCs and LSCs. Importantly, the phenotype of *Light* and *Ltbr* single KO HSCs was similar to *Light/Ltbr* double KO HSCs after treatment with 5-FU, excluding a major contribution of other receptors in the maintenance of quiescence. As many other members of the TNF superfamily (e.g., TNF-α, Fas-L), LIGHT can act in a soluble or transmembrane form. Importantly, soluble and transmembrane isoforms of TNF superfamily members, including LIGHT, cause different effects on various cell types^67–69^. We now document that neither soluble LIGHT protein nor transmembrane LIGHT expressed by another cell, did rescue the phenotype of *Light*^−/−^ HSCs, indicating that cell-autonomous LIGHT/LTβR signaling is responsible for the observed effects.

LIGHT/LTβR signaling maintains stemness by reducing cell proliferation and favoring symmetric over asymmetric cell division. We analyzed the cell division pattern in most experiments by defining dividing cells and analyzing Numb distribution according to the cleavage furrow. This analysis has been used by many groups before^63,70,71^, but does not allow to precisely define the axis of cell division. Importantly, the inclusion of an α-tubulin staining confirmed an increased AD in *Ltbr*^−/−^ HSPCs, suggesting that LTβR signaling regulates the cell division pattern. Many molecular pathways that regulate stemness and differentiation also regulate apoptosis and vice versa^72^. In the present study, we documented a reduced apoptosis rate in *Ltbr*^−/−^ HSCs that further contributes to the initial cell expansion of more differentiated cells.

It has been shown before that an increase of asymmetric cell division leads to differentiation and exhaustion of stem cells^12,73,74^. This is reflected in an increase in colony formation capacity in first platings and in an improved engraftment after transplantation into first recipient mice, a process that is mediated by both HSCs and progenitors^32^. The exhaustion of functional HSCs leads then to a reduced engraftment in second and third re-platings or re-transplantations.

The absence of LTβR signaling induced an upregulation of genes involved in cell cycling and proliferation such as *Cdk4* and *Cdk6*. These cyclin-dependent kinases are known to complex with cyclin D1 to regulate G1/S transition and the exit of HSCs from quiescence^75,76^. Accordingly, *Ltbr*^−/−^ HSCs showed a positive correlation with the signature of positive regulation of G1−S transition in GSEA analysis. Similarly, knockdown of *LTBR* in human CD34^+^ HSPCs increased the expression of genes involved in cell cycling while reducing genes associated with stemness. Functional analysis of HSCs confirmed a reduced proliferation of LIGHT/LTβR-competent stem cells, especially of the most primitive LT-HSCs. Moreover, we identified a similar role for LTβR in the maintenance and expansion of CML stem cells. *Ltbr-* and *Light*-knockout in LSCs reduced the number of leukemia HSPCs in the BM and prolonged survival in a murine CML model. Importantly, mouse and human CML cells overexpress *LTBR* and *LIGHT* when compared to normal HSPCs. Knockdown of *LTBR* in human CML HSPCs resulted in the downregulation of stemness- and Wnt-related genes, such as *MSI2*, *TNIK*, and *CTNNB1* and a reduced number of LSCs that form colonies in methylcellulose. Since LSCs are resistant to chemotherapy and probably also TKIs, targeting pathways that regulate and maintain LSCs will be crucial to improve the treatment of leukemia^77,78^. Importantly, LTβR deficiency did not affect steady-state hematopoiesis but did only regulate HSC quiescence and cell fate in demand-adapted hematopoiesis. Thus, targeting the LIGHT/LTβR pathway may offer a novel strategy to induce differentiation and to eliminate LSCs.

## Methods

### Animals

BL/6 and Ly5.1 mice were purchased from Charles River. *Ltbr*^−/−^ mice were kindly provided by Prof. A. Aguzzi (Institute of Neuropathology, University Hospital of Zurich, Switzerland; Futterer et al.^22^) and *Light*^−/−^ mice by S. Scheu (University Hospital of Düsseldorf, Germany). KO strains are homozygote mutant mice, generated by intragenic deletions^22,23^. Intercrosses were validated by genotyping PCR and subsequently used for animal husbandry. *Light*^−/−^ mice on Ly5.1 background were referred to as Ly5.1/*Light*^−/−^ mice. Offspring mice from Ly5.1×BL/6 breeding are used as recipient mice (referred to as Ly5.1/Ly5.2). C57BL/6-Tg(UBC-GFP)30Scha/J mice were kindly provided by Dr. Mario Bonalli (LASC, University of Zurich, Switzerland)^79^. Mice aged 6–13 weeks were used for experiments. Animal experiments were approved by the local experimental animal committee of the Canton of Bern and performed according to Swiss laws for animal protection.

### BM and blood samples from patients

BM samples from untreated staging negative lymphoma patients, who were considered normal by a surgical pathologist and a hematologist, were used as control samples. HSPCs were isolated from apheresis samples of patients that have been treated with chemotherapy and G-CSF. PB samples and BM aspirates from untreated CML patients were taken at diagnosis. Patient samples were collected at the University Hospital of Bern after written informed consent. Patient characteristics are listed in Supplementary Tables 1–3. Analysis of samples was approved by the local ethical committee of the Canton of Bern (KEK122/14).

### Isolation of BM cells and lineage depletion

Mice were sacrificed; femurs, tibiae, humeri, and the spine were crushed in phosphate-buffered saline (PBS). The BM suspension was filtered through a 40-µm cell strainer. Subsequently, red blood cell lysis was performed and cells were washed twice with PBS. Lineage depletion was performed with MACS separation according to the manufacturer’s protocol. Briefly, BM cells were stained with biotinylated antibodies (all BioLegend, San Diego, USA) against red cell precursors (αTer119), B cells (αCD19), T cells (αCD3ε), myeloid cells (αGr1), and α-biotin MicroBeads using LS columns (Miltenyi Biotec, Bergisch Gladbach, Germany).

### Isolation of BM niche cells

BM MSCs, ECs, and osteoblasts were isolated according to Schepers et al.^80^. Briefly, bones were cleaned, crushed, and digested with collagenase and DNase. Isolated cells were stained with αCD31^−^FITC (clone:390) αCD51-APC (clone:RMV-7) αLy-6A/E-PerCP-Cy5.5 (clone:D7, all BioLegend, San Diego, USA) and Streptavidin-V500 (BD Bioscience, Eysins, Switzerland) and hematopoietic cells were excluded using anti-lineage antibodies (αTer119, αCD19, αCD3ε and αGr1, αCD45 (clone:30F11)). BM niche cells were characterized using the following surface markers: EC, CD45^−^CD31^+^sca1^+^; MSC, CD45^−^Lin^−^CD31^−^sca1^+^CD51^+^; and osteoblasts, CD45^−^Lin^−^CD31^−^sca1^−^CD51^+^.

### Homing assay

Ly5.1 and *Ltbr*^−/−^ LSKs were FACS purified and 26,000 to 54,000 LSKs were injected at a ratio of 1:1 into lethally irradiated Ly5.1/Ly5.2 recipients. Thirteen hours post transplantation the frequency of LSKs homed to the BM cells was analyzed by FACS. The frequency of Lin^−^CD45.1^+^ and Lin^−^CD45.2^+^ cells was calculated as the percentage of transplanted LSKs.

### Treatment with 5-FU

BL/6, Ly5.1, and *Ltbr*^−/−^ mice were injected intraperitoneally with 150 mg/kg 5-FU (Sigma). Eight to 9 days postinjection, BM cells from Ly5.1 and *Ltbr*^−/−^ mice were analyzed or injected at a ratio of 1:1 into lethally irradiated Ly5.1/Ly5.2 recipients (2 × 6.5 Gy with 4-h interval, Gammacell 40 exactor, Best Theratronics, Ottawa, Canada).

### Generation of chimeric mice

A total of 15,000–20,000 LSKs from Ly5.1, *Ltbr*^−/−^, or *Light*^−/−^ mice were injected intravenously at a ratio of 1:1 into lethally irradiated Ly5.1/Ly5.2 or Ly5.1/*Light*^−/−^ recipient mice. Serial re-transplantation experiments were performed 12–18 weeks after primary transplantation. Ly5.1 and *Ltbr*^−/−^ LSKs from chimeras were FACS purified and injected intravenously (i.v.) at a ratio of 1:1 into lethally irradiated Ly5.1/Ly5.2 recipient mice. If re-transplantation was done with <5000 LSKs (third, fourth transplantation) rescue BM cells were given.

### CML mouse model

CML was induced in mice^45^. Briefly, FACS-purified LSKs were plated in RPMI medium, supplemented with 10% fetal calf serum (FCS), 1% glutamine, 1% penicillin/streptomycin, 20 ng/ml thrombopoietin, 100 ng/ml SCF overnight, and subsequently transduced by spin infection with BCR-ABL1-GFP retrovirus. CML was induced by i.v. injection of 30,000 transduced LSKs into nonirradiated recipient mice (BL/6 in BL/6: BL/6 CML; *Ltbr*^−/−^ in BL/6: *Ltbr*^−/−^ CML; *Light*^−/−^ in BL/6: *Light*^−/−^ CML). Blood counts were analyzed using a Vet abc Animal Blood Counter (Medical Solution GmbH, Bassersdorf, Switzerland). Mice were analyzed at days 18–20 after CML induction. For secondary transplantations, 5 × 10^6^ BM cells or 20,000 FACS-purified LSCs from primary CML mice (18–20 days after primary transplantation) were injected i.v. into secondary immunocompetent recipients.

### Cell cycle analysis

Ki67 staining was performed with Foxp3/Transcription Factor Staining Buffer Set (eBioscience, San Diego, USA) according to the manufacturer’s protocol. In addition, cells were stained with DAPI (Merck, Darmstadt, Germany) and analyzed by FACS. BrdU incorporation was determined according to the manufacturer’s protocol (BD Pharmingen BrdU Flow Kits, San Jose, USA).

### ImageStream analysis

Numb/α-tubulin (Numb: ab4147, Tubulin: ab7291, Abcam, Cambridge, UK) staining in FACS-purified LSKs or LT/ST-HSCs from chimeric mice, 5-FU-treated BL/6 and *Ltbr*^−/−^ mice (8 days after treatment) or CML mice was performed as follows: cells were fixed with 4% paraformaldehyde, followed by permeabilization with 1× wash buffer (Dako wash, Agilent Technologies, California, USA) and blocking with 10% normal goat serum (Invitrogen, California, USA) in Dako wash. After overnight incubation at 4 °C with the primary rabbit α-Numb antibody or tubulin in Dako diluent, cells were incubated with the secondary antibody (donkey-anti-goat, ab175474; goat-anti-mouse, ab150115) for 1 h at room temperature^63^. DAPI was used to stain for DNA. Asymmetric cell division was determined by an increase in Numb intensity of 1.8-fold in one of the daughter cells^11^. Cells were acquired using an ImageStreamX MkII imaging flow cytometer (Merck, Darmstadt, Germany). Cells were analyzed using INSPIRE and IDEAS Software^63^.

### Colony-forming cell assays

FACS-purified BM LSKs (600–1 × 10^3^ naive LSKs; 1 × 10^4^ 5-FU-treated Lin^−^sca1^+^) were plated in MethoCult (STEMCELL Technologies, Cambridge, USA) supplemented with 15% FCS, 20% BIT (50 mg/ml bovine serum albumin in Iscove’s modified Dulbecco’s medium), 1.44 U/ml rh-insulin and 250 ng/ml human holo-transferrin, 100 µM 2-mercaptoethanol, 100 U/ml penicillin, 100 µg/ml streptomycin, 2 mM l-glutamine, 50 ng/ml SCF (rmSCF-1), 10 ng/ml interleukin-3 (rmIL-3), 10 ng/ml interleukin-6 (rhIL-6), and 50 ng/ml fms-related tyrosine kinase 3 ligand (rmFLTL-3). Re-plating was performed with 10,000 cells per dish. For CFU assays of human HSPCs, we plated 1000 CD45^int^Lin^−^CD34^+^ BM cells into methylcellulose. Colonies were enumerated after 7–14 days in culture (≥30 cells/colony) on a DMIL inverted microscope (Leica, Wetzlar, Germany)

### Flow cytometry and cell sorting

Cells were stained in PBS with 5% FCS with the following antibodies for 30 min at 4 °C: αCD117-APC-Cy7 (clone:2B8), αCD48-PE-Cy7 (clone:HM48-1), αCD150-APC (clone:TC1512F12.2), αLy-6A/E-PerCP-Cy5.5, -APC (clone:D7, eBioscience, San Diego, USA), αCD16/CD32-PE-Cy7 (clone:93), αCD34-eFluor-450 (clone:RAM34, eBioscience, San Diego, USA), αCD135-PE, -biotin (clone:A2F10, Novus, Littleton, USA), αCD127-FITC (clone:A7R34), αCD45.1-PerCP-Cy5.5 (clone:A20), αLTβR-PE (clone:ebio3C8, eBioscience, San Diego, USA), αLy-6C-PerCP-Cy5.5 (clone:HK1.4), αCD11b-PE-Cy7, αCD8a-FITC, -APC, FITC (clone:53-6.7), αCD4 (clone:GK1.5), αCD90.1-APC (clone:OX-7), αCD90.2-APC (clone:30-H12), Annexin-V-Pacific-Blue, -Alexa Fluor 647, PE, αLy-6G-Pacific-Blue, αCD45.2-Alexa Fluor 700 (clone:104), Ki67-PE (clone:16A8), rat IgG2a,κ-PE (clone:RTK2758), αCD45-PerCP-Cy5.5, -PE-Cy7, -APC (clone:30F11). Human HSPCs were stained with αCD90-PeCP-Cy5.5 (clone:5E10), αCD34-APC (clone:561), αLTβR-PE (clone:31G4D8), αCD38^−^APC (clone:HIT2), mouse-IgG2b,κ (clone:MPC-11), αCD2-biotin (clone:RPA2.10), αCD3Ɛ-biotin (clone:OKT3), αCD14-biotin (clone:HCD14), αCD16-biotin (clone:3G8), αCD19-biotin (clone:HIB19), αCD56-biotin (clone:HCD56), αCD235a-biotin (clone:HIR2), αCD45-Pacific-Blue (clone:2D1), Streptavidin-V500 (all from BioLegend, San Diego, USA). For intracellular staining of NF-κB members, cells were surface stained, followed by 4% paraformaldehyde fixation and permeabilization via ice-cold methanol, according to the manufacturer’s protocol. Following primary antibodies were used: phospho-IκBα (Ser32) (clone:14D4), IκBα (clone:44D4); secondary antibody: anti-rabbit IgG (H + L), F(ab′)2 Fragment-Alexa Fluor 647 (all from Cell Signaling Technology Inc., Massachusetts, USA). FACS was performed on a LSR Fortessa cell analyzer or LSRII Flow cytometer (both BD Bioscience, San Jose, USA). Cell sorting was done using a BD FACSARIA III (BD Bioscience, San Jose, USA). Data analysis was performed with Kaluza Flow analysis software (Beckman Coulter, Krefeld, Germany) or FlowJo software (Treestar, Oregon, USA).

### RNA isolation and quantitative RT-PCR

RNA was isolated according to the manufacturer’s protocol (NucleoSpin RNA XS, Macherey-Nagel, PA, USA) and the expression of genes was analyzed using SYBR Green 2× PCR Master Mix (Roche, NY, USA) on a 7500 real-time PCR System (AB Biosystems, CA, USA). *Actin* or *Gapdh* genes were used for normalization of the gene expression. The sequences of primers are listed in Supplementary Table 4.

### High-throughput transcriptome sequencing (RNA-seq)

Total RNA was extracted from FACS-purified *Ltbr*^−/−^ and Ly5.1 HSCs (Lin^−^Annexin-V^−^sca1^+^c-kit^+^CD150^+^CD48^−^) from chimeric mice using the RNeasy Micro Kit (Qiagen AG, Switzerland) according to the manufacturer’s instructions. Total RNA was quality checked on the Bioanalyzer instrument (Agilent Technologies, USA) using the RNA 6000 Pico Chip (Agilent, USA) and quantified by Fluorometry using the QuantiFluor RNA System (Promega, USA).

Library preparation (average library size: 313 ± 3 bp) was performed from 20 ng total RNA using the TruSeq Stranded mRNA Library Prep Kit High Throughput (Cat# RS-122-2103, Illumina, San Diego, CA, USA) and quality (average concentration: 6.7 ± 1.8 nmol/L) was analyzed on the Fragment Analyzer (Advanced Analytical, Ames, IA, USA, High Sensitivity NGS Fragment Analysis Kit, Cat# DNF-473, Advanced Analytical). Samples were pooled, quantified, adjusted to equal molarity (1.4 pM, QuantiFluor ONE dsDNA System, Cat# E4871, Promega, Madison, WI, USA), and used for clustering on the NextSeq 500 instrument (Illumina). Samples were sequenced in single reads with 76 bases using the NextSeq 500 High Output Kit 75 cycles (Illumina, Cat# FC-404-1005). Primary data analysis was performed with the Illumina RTA version 2.4.11 and Basecalling Version bcl2fastq-2.20.0.422. An average per sample of 62 ± 3.6 million reads was obtained.

### RNA-seq data analysis to access differentially expressed genes

The RNA-seq data were assembled by SeqMan NGen software v.16 and analyzed using ArrayStar software v.16 (DNASTAR, USA). The level of gene expression was assessed using regularized logarithm values from Bioconductor. After statistical analysis, genes with a significant difference in their expression at false discovery rate *p* < 0.05 and fold differences ≥1.5 were selected. Data were clustered using standard Euclidean’s method based on the average linkage and heatmaps were generated according to the standard normal distribution of the values (Supplementary Dataset 1).

### Gene set enrichment analysis

GSEA was performed using GSEA software v.3.0 (Broad Institute). Enrichment analysis was assessed for all pathway-related genes acquired from PathCards database (pathcards.genecards.org).

### Transfection of human HSPCs with siRNA

FACS-purified BM CD34^+^ HSPCs from patients undergoing a diagnostic BM aspirate that was considered normal by a hematologist and an independent surgical pathologist (control donor) and CD34^+^ HPSCs from the blood of G-CSF-treated patients were cultured in StemSpan SFEM medium in combination with StemSpan CC100 (STEMCELL Technologies) and transfected with control siRNA (siCTRL), *LTbR* targeting siRNA (si*LTBR*), or *LIGHT* targeting siRNA (si*LIGHT*) (Santa Cruz Biotechnology, Heidelberg, Germany) using TransIT-X2 (Mirus Bio Muttenz, Switzerland) according to the manufacturer’s protocol. Genes were analyzed 24 h post transfection unless otherwise stated.

### Statistical analysis

Statistics were calculated using Prism 8.0 (Graph Prism Software, USA). Data were analyzed using one-way analysis of variance and Tukey’s or Dunnett’s multiple comparison test or Student’s *t* test. Survival curves were analyzed using a log-rank (Mantel–Cox) test. Data are displayed as mean ± SEM. *P < 0.05, **P < 0.01, ***P < 0.001, and ****P < 0.0001.

### Reporting summary

Further information on research design is available in the Nature Research Reporting Summary linked to this article.

## Supplementary information

Supplementary Information

Reporting Summary

Description of Additional Supplementary Files

Supplementary Data 1

## Data Availability

All relevant data are available upon request from the authors. All transcriptomic data compiled for this study have been deposited in NCBI GEO under the accession code: GSE141206. Expression data were derived from a public repository for microarray data (GEO) and are available under accession number GSE32719 (ref. ^46^) and GSE11675 (ref. ^47^).
