# Supplementary material for: LIGHT/LTβR signaling regulates self-renewal and differentiation of hematopoietic and leukemia stem cells
Source: Nat Commun. 2021 Feb 16;12:1065. doi: 10.1038/s41467-021-21317-x (PMC7887212; doi:10.1038/s41467-021-21317-x)
Supplement: Supplementary file 1 — Supplementary Information [file 41467_2021_21317_MOESM1_ESM.pdf]

LIGHT/LTβR signaling regulates self-renewal and differentiation of hematopoietic and leukemia stem cells

Höpner, S.S et al.

- Supplementary material and methods
- Supplementary Figures 1-8
- Supplementary Tables 1-4

Supplementary information

Supplementary material and methods

Gene ontology (GO)

For GO enrichment, the list of differently expressed genes was grouped into functional hierarchies. Enrichment scores were calculated using a chi-square test comparing the proportion of the gene list in a group to the proportion of the background genes. A value ≥ 3 corresponded to a significant over-expression ($p<0.05$).

Gene expression profiling using Fluidigm system

Gene expression profiling was performed with FACS-purified LSKs from chimeric mice 6 weeks after transplantation using a Fluidigm®96.96 Dynamic Array™ on the BioMark system (Fluidigm, San Francisco, United States) according to the manufacturer's protocol. Assays were designed based on EvaGreen® chemistry and primers for targeting desired pathways were designed accordingly for amplicons of 100-160bp using Primer3Plus (Supplementary Table 4). [1] Briefly, total RNA was isolated from FACS-purified LSKs 6 weeks post-transplantation using RNeasy® Micro Kit (QIAGEN AG, Basel, Switzerland). The quantity of extracted RNA was assessed using a NanoDrop ND-1000 spectrophotometer (Biolab, Mulgrave, VIC, Australia). For each sample, the extracted RNA was used to synthesize cDNA using the High Capacity cDNA Reverse Transcription Kit (AB™, CA, United States). cDNA was pre-amplified with a mix of primers specific to the target genes as the specific target amplification (STA) for 15 cycles. STA products were then diluted fivefold and analyzed with TaqMan™ Gene Expression Master Mix and EvaGreen DNA binding dye in a 96:96 Fluidigm dynamic array. Ct values were calculated and visualized using BioMark real-time PCR analysis software (Fluidigm, San Francisco, United States). Each assay was performed in replicate.

Genes with Ct values of higher than 35.0 or differences of ≥1.0 in-between sample replicates were eliminated from the analysis. If the reference genes (*Actb* and *Gapdh*) were not expressed or differences of ≥1.0 in-between sample replicates were observed, the sample was not included in the analysis. Raw values were normalized using the geometric mean of two reference genes (*Actb* and *Gapdh*). The fold difference for each sample was calculated using the comparative Ct method.[2] Relative gene expression quantities after log2 transformation were used for data analysis. Data were clustered using standard Euclidean's method based on the average linkage.

Cell signaling and *in silico* pathway analysis

Gene networks representing differentially expressed genes were identified using the Ariadne Genomics Pathway Studio® database (Elsevier, Berlin, Germany).

Supplementary Figures

Supplementary Figure 1

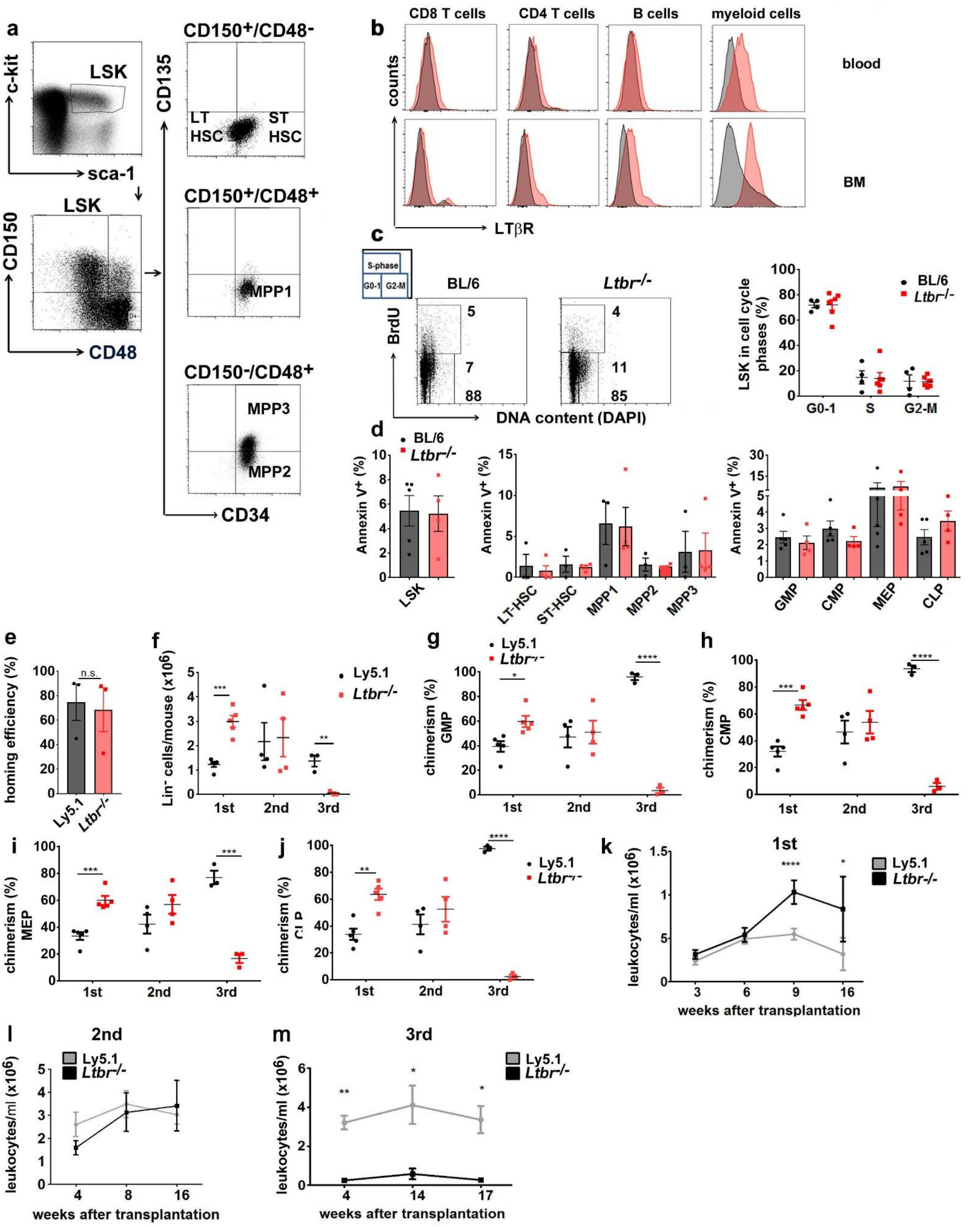

Supplementary Figure 1: LTβR is dispensable in steady state hematopoiesis but is crucial for reconstitution after hematopoietic stress. (a) Gating strategy of LSK subsets (LT-HSC, ST-HSC, MPP1, MPP2, MPP3). **(b)** Representative histogram of LTβR expression on indicated differentiated cells in blood and BM of naïve BL/6 mice. (grey: isotype control, red: LTβR staining). **(c)** Cell cycle analysis of naïve BL/6 (n=4) and *Ltbr*$^{-/-}$ LSKs (n=7). Representative FACS dot plots and the percentage of cells in G0-1, S and G2-M phase are shown. **(d)** Percentages of Annexin-V^{+} LSKs, LSK subsets (BL/6 n=3, *Ltbr*$^{-/-}$ n=4) and progenitor subsets in the BM of naïve BL/6 and *Ltbr*$^{-/-}$ mice (BL/6 n=5, *Ltbr*$^{-/-}$ n=4). **(e)** Homing of Ly5.1 and *Ltbr*$^{-/-}$ LSKs to the BM (% of CD45.1 and CD45.2 LSKs in the BM of Ly5.1/Ly5.2 mice relative to the numbers of total injected LSKs, (n=3). **(f)** Cell numbers of Ly5.1 and *Ltbr*$^{-/-}$ BM Lin^{-} cells in 1st, 2nd and 3rd transplantation, n=5 (1st), n=4 (2nd), n=3 (3rd) mice.**(g-j)** Percentage of Ly5.1 and *Ltbr*$^{-/-}$ GMPs (g), CMPs

(h), MEPs (i) and CLPs (j) in 1st, 2nd and 3rd transplantation. Data are shown as mean±SEM of n=5 (1st), n=4 (2nd), n=3 (3rd) mice. **(k-m)** Numbers of Ly5.1 and *Ltbr*$^{-/-}$ leukocytes in Ly5.1/Ly5.2 recipients at indicated time points after 1st (k), 2nd (l) and 3rd (m) transplantation, n=5 (1st), n=4 (2nd), n=3 (3rd) mice. Data are depicted as mean±SEM. Statistics: * P<0.05, ** P<0.01, *** P<0.001 (two-tailed *t*-test). 1f: 1st p=0.0002, 3rd p=0.0048; 1g: 1st p=0.012, 3rd p<0.0001; 1h: 1st p=0.0002, 3rd p<0.0001; 1i: 1st p=0.0003, 3rd p=0.0006; 1j: 1st p=0.001, 3rd p<0.0001; 1k wk9 p<0.0001, wk16 p=0.032; 1m: wk4 p=0.0012, wk14 p=0.026, wk17 p=0.012.

Supplementary Figure 2:

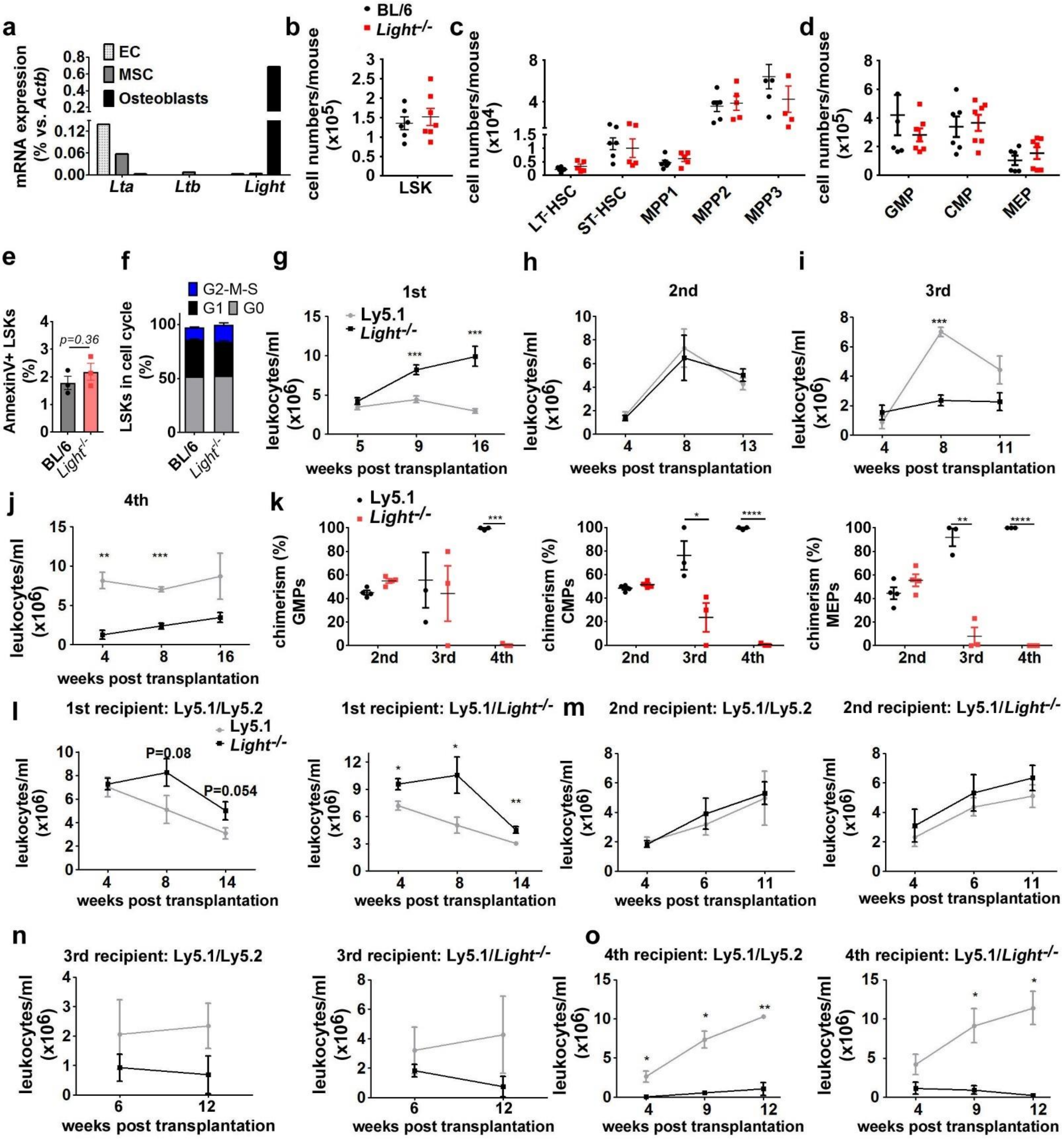

Supplementary Figure 2: Cell-autonomous LIGHT expression prevents exhaustion of HSCs.

(a) Relative expression of *Light*, *Lta* and *Ltb* of FACS-purified EC; MSC and Osteoblasts from naïve BL/6 mice (cells were pooled from 10 mice). **(b-d)** Numbers of LSKs (b), LSK subsets (LT-HSC, ST-HSC, MPP1, MPP2, MPP3) (c) and myeloid progenitors (GMP, CMP, MEP) (d) in the BM of naïve BL/6 and *Light*$^{-/-}$ mice (BL/6 n=6, *Light*$^{-/-}$ n=7). **(e)** Percentages of Annexin-V^{+} LSKs of BL/6 (n=3) and *Light*$^{-/-}$ (n=3) mice. Data are shown as mean±SEM. **(f)** Percentage of BL/6 (n=2) and *Light*$^{-/-}$ (n=3) LSKs in G0-1, S and G2-M phase. Data are shown as mean±SEM **(g-j)** Numbers of BL/6 and *Light*$^{-/-}$ leukocytes in blood from Ly5.1/Ly5.2 recipients at the time points indicated post transplantation, 1st (g), 2^{nd}(h), 3rd (i), 4^{th}(j). **(k)** Percentage of BL/6 and *Light*$^{-/-}$ myeloid progenitors (GMP, CMP, MEP) in the 2nd, 3rd and 4th transplantation. Data are shown as mean±SEM of n=4 (2nd), n=3 (3rd, 4th) mice. **(l-o)** Number of donor leukocytes in blood of Ly5.1/Ly5.2 and congenic Ly5,1/*Light*$^{-/-}$ recipients in 1st (l), 2nd (m), 3rd (n) and 4th (o) serial transplantation. Data are shown as mean±SEM, Ly5.1/Ly5.2 recipients: n=7 (1st), n=5 (2nd), n=3 (3rd); n=3 (4th) Ly5.1/*Light*$^{-/-}$ recipients: n=5 (1st), n=5 (2nd), n=3 (3rd), n=3 (4th). Statistics: * $P<0.05$, ** $P<0.01$, *** $P<0.001$, **** $P<0.0001$ (two-tailed *t*-test). 2g: 1st wk9 p=0.0003, wk16 p=0.0002; 2i: 3rd wk8 p=0.0006; 2j:4th wk4 p=0.004, wk8 p=0.0006; 2k: GMP p=0.0092, CMP 3rd p= 0.038, 4th p<0.0001; MEP 3rd p=0.0014, 4th p<0.0001; 2m: Ly5.1/*Light*$^{-/-}$ wk4 p=0.023, wk8 p=0.03, wk12 p=0.006; 2o:Ly5.1/Ly5.2 wk4 p=0.02, wk9 p=0.03, wk12 p=0.02, Ly5.1/*Light*$^{-/-}$ wk9 p=0.025, wk12 p=0.034.

Supplementary Figure 3:

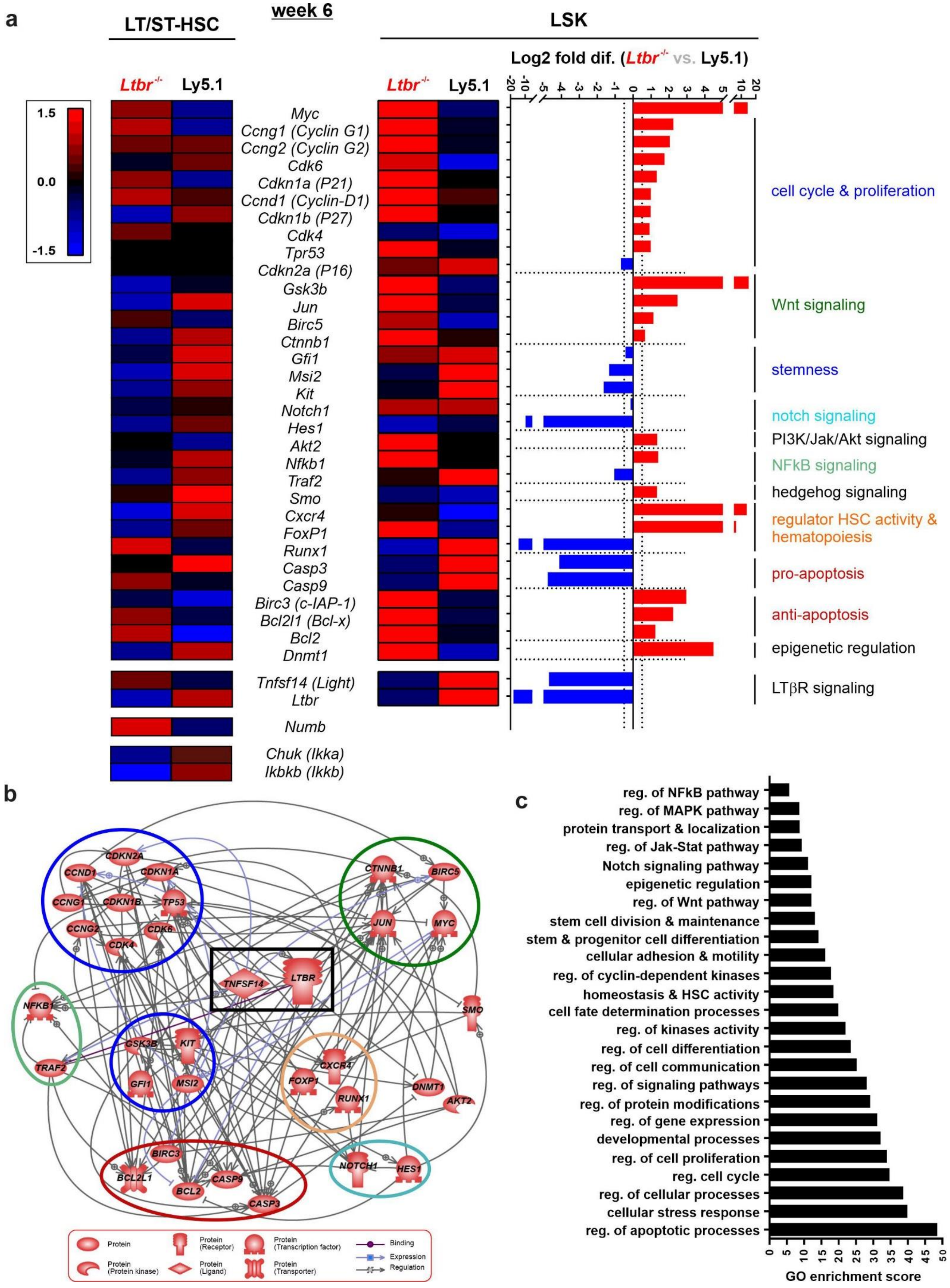

Supplementary Figure 3: Gene expression analysis for LT-HSCs and LSKs from chimeric mice

(a) Heatmap of differentially expressed genes in FACS-purified *Ltbr*$^{-/-}$ LT/ST-HSCs (Lin^{-}, c-kit^{+}, sca-1^{+}, CD150^{+}, CD48^{-}, left panel) and LSKs (right panel) compared to Ly5.1 LT/ST-HSCs and LSKs respectively from chimeras six weeks after primary transplantation (gene expression log2 fold differences *Ltbr*$^{-/-}$ vs. Ly5.1; n=4). **(b)** *In silico* gene network and canonical pathway analysis of the differentially expressed genes in LSKs; colors of the circles are corresponding to pathways highlighted in part a. **(c)** Gene ontology (GO) enrichment analysis of the biological pathways regulated by LTβR expressed on LSKs.

Supplementary Figure 4:

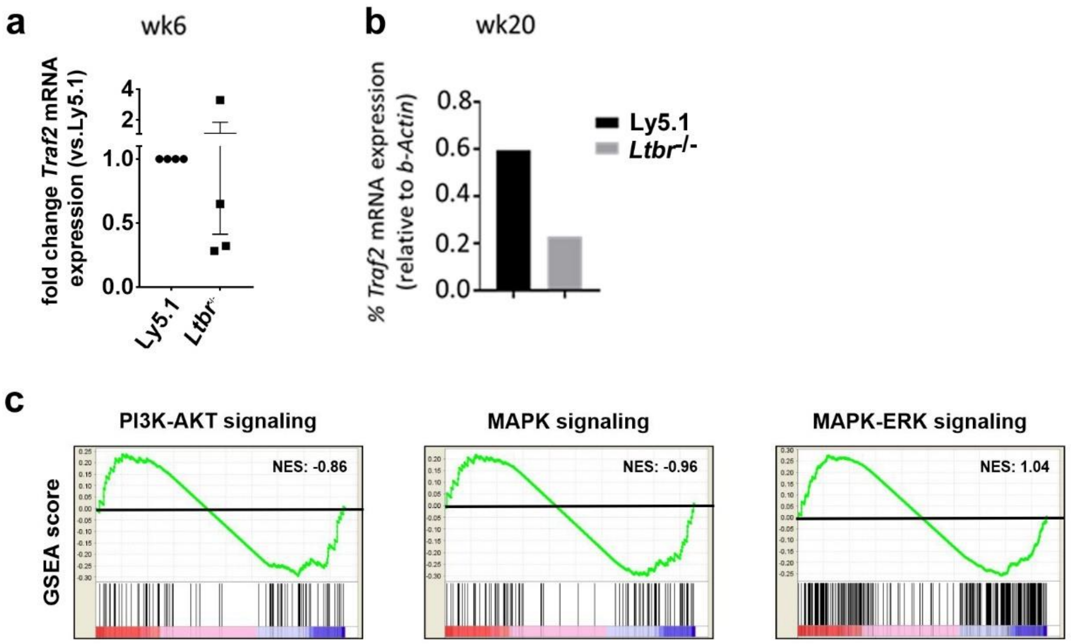

Supplementary Figure 4: analysis of signaling pathways involved in LIGHT-mediated activation

(a) Fold change of *Traf2* mRNA expression of *Ltbr*$^{-/-}$ LSKs relative to the expression in Ly5.1 LSKs from chimeras 6 weeks post-transplantation, n=4. Data are shown as mean±SEM. **(b)** Relative *Traf2* mRNA expression of Ly5.1 and *Ltbr*$^{-/-}$ LSKs from chimeras 20 weeks post-transplantation, cells for analysis are pooled from n=4 mice. **(c)** Gene set enrichment analysis of *Ltbr*$^{-/-}$ LT/ST-HSCs from chimeric mice 6 weeks post-transplantation for signatures associated with PI3K-AKt, MAPK and MAPK-ERK signaling.

Supplementary Figure 5:

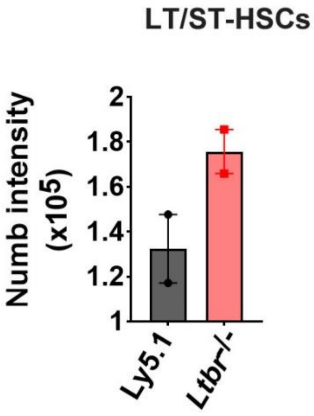

Supplementary Figure 5: Analysis of Numb expression on LT/ST-HSCs

Numb intensity (MFI) of *Ltbr*$^{-/-}$ and Ly5.1 LT/ST-HSCs from chimeric mice 10-12 weeks post-transplantation (pooled data from n=9 mice in two independent experiments). Data are presented as mean±SEM.

Supplementary Figure 6:

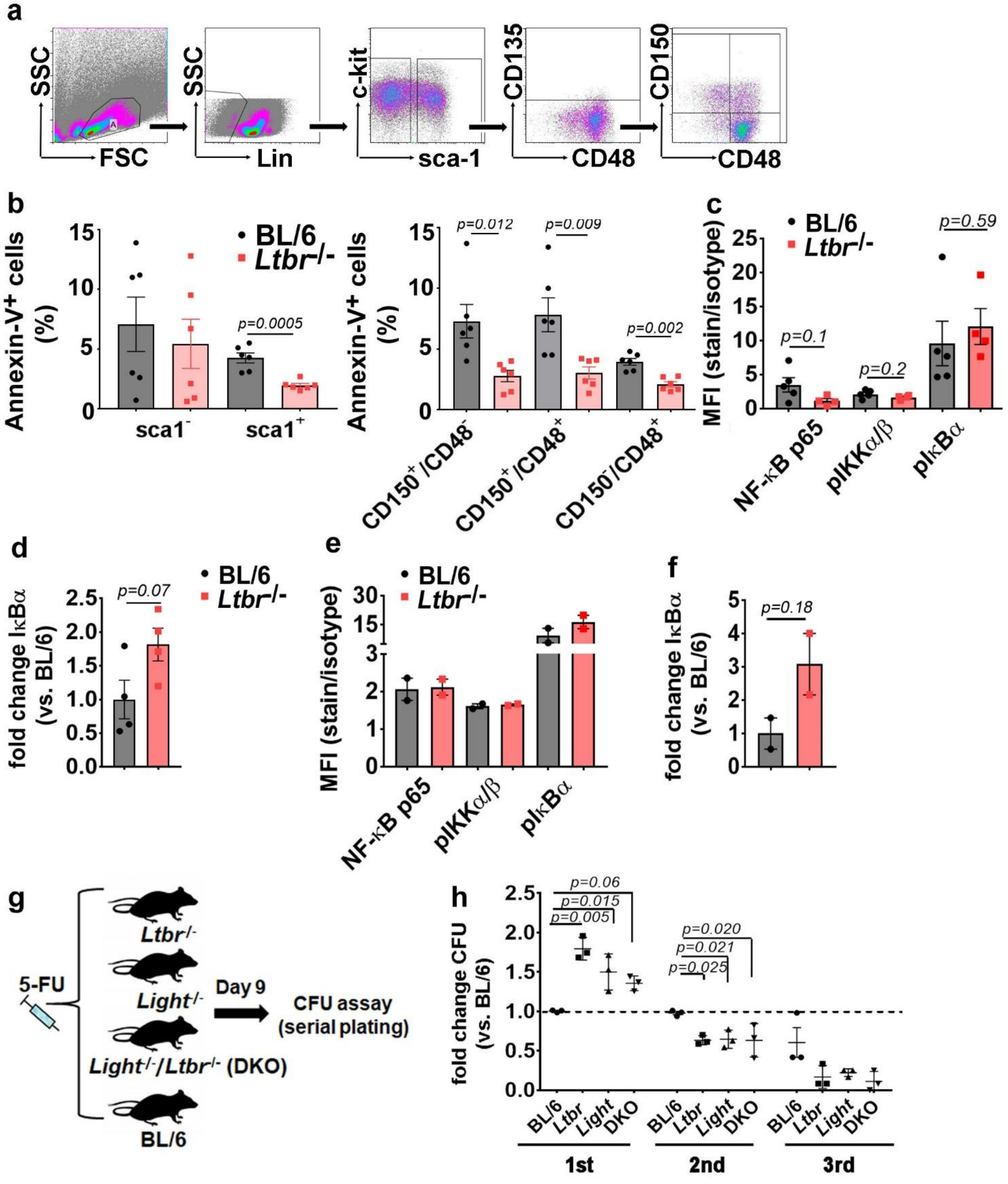

Supplementary Figure 6: Analysis of cell death and colony formation of 5-FU treated mice

(a) Gating strategy of LSK subsets (LT-/ST-HSCs, MPP1, MPP2/3) from mice treated with 150mg/kg 5-FU, eight days post-infection, accordingly to the expression of c-kit and sca-1. **(b)** Percentage of apoptotic sca1^{-} and sca1^{+} cells within BL/6 and *Ltbr*$^{-/-}$ BM Lin^{-} cells, eight days after 5-FU treatment and percentage of Annexin-V^{+} in LSK subsets from BL/6 and *Ltbr*$^{-/-}$ mice. One out of two independent experiments is shown (n=6 mice, per group). **(c)** Protein expression (MFI) of NFκBp65, phosphorylated IKKα/β and IκBα of naïve BM LT/ST-HSCs from BL/6 n=5 and *Ltbr*$^{-/-}$ n=4 mice. Data are pooled for analysis from two independent experiments. **(d)** Fold change of total IκBα in *Ltbr*$^{-/-}$ BM LT/ST-HSCs relative to BL/6. Data are pooled for analysis from two independent experiments, BL/6 n=4 and *Ltbr*$^{-/-}$ n=4 mice. **(e)** Protein expression (MFI) of NFκBp65, phosphorylated IKKα/β and IκBα of BM LT/ST-HSCs (Lin^{-}sca^{-}1^{+}CD48^{-}CD150^{+}) from BL/6 and *Ltbr*$^{-/-}$ mice eight days post-treatment with 5-FU, n=2 mice for each data point. One out of two independent experiments is shown. **(f)** Fold change of total IκBα in BM LT/ST-HSCs from 5-FU treated *Ltbr*$^{-/-}$ mice relative to BM LT/ST-HSCs from 5-FU treated BL/6 mice, n=2 mice for each data point. One out of two independent experiments is shown. Data are presented as mean±SEM. **(g)** Schematic of 5-FU treatment for *Light*$^{-/-}$/ *Ltbr*$^{-/-}$ double knockout (DKO), *Light*$^{-/-}$, *Ltbr*$^{-/-}$ and BL/6 mice. **(h)** Fold change of CFU capacity of FACS-purified BL/6, *Ltbr*$^{-/-}$, *Light*$^{-/-}$ and DKO LSKs in serial re-plating experiments, (n=3). Data are presented as mean±SEM. Statistics: two-tailed *t*-test.

Supplementary Figure7:

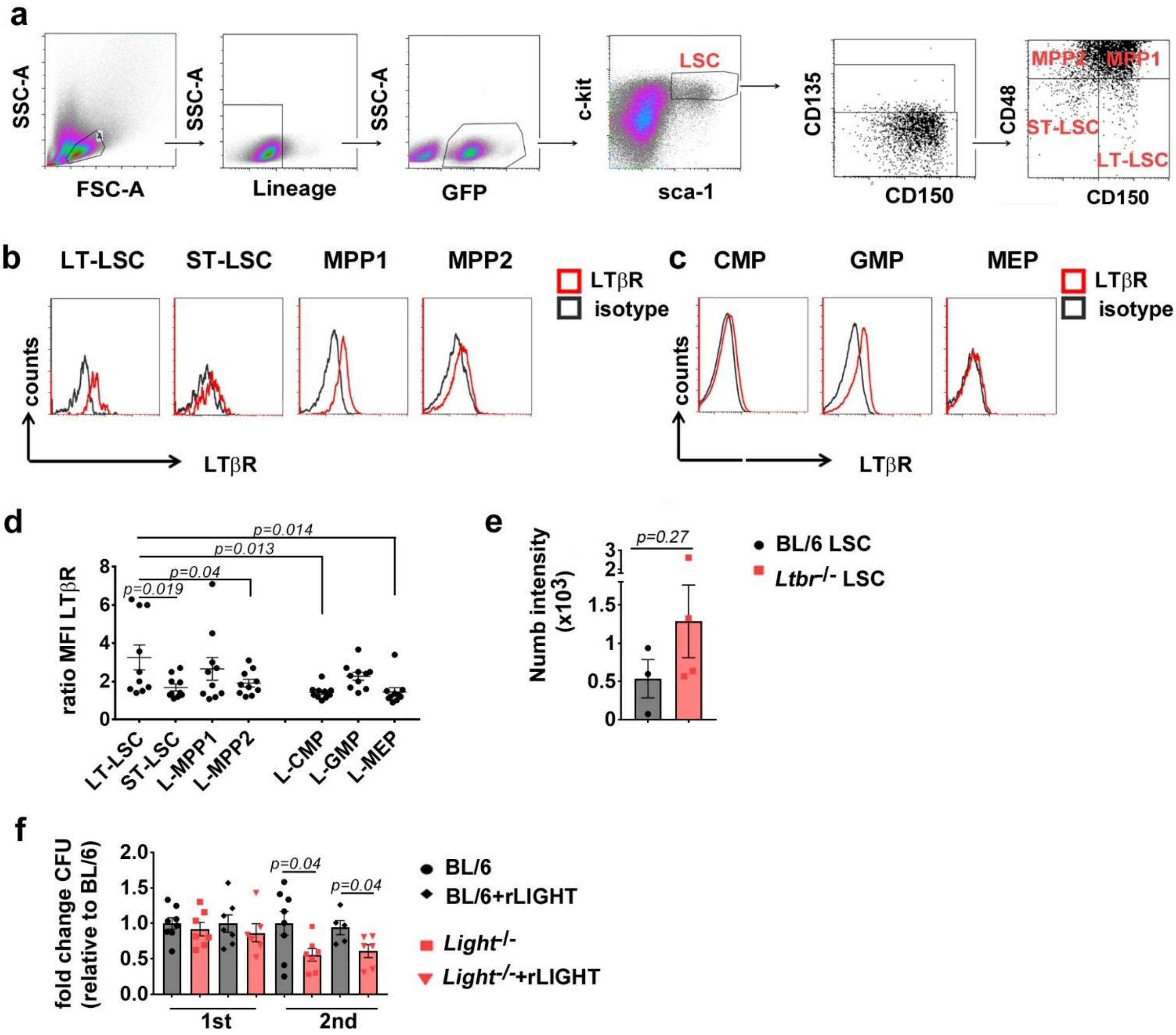

Supplementary Figure 7: LTβR signaling in CML bearing mice.

(a) Gating strategy of LSCs and LSC subsets in CML mice. **(b-c)** Representative histograms of LTβR expression on LSC subsets (b) and BM myeloid progenitors (c) of BL/6 CML mice 19 days after CML induction. **(d)** Quantification of LTβR expression in BL/6 LSCs, LSC subsets and myeloid progenitors from BL/6 mice (ratio MFI: MFI stain/ isotype, n=10). **(e)** Numb intensity (MFI) of BL/6 and *Ltbr*$^{-/-}$ LSCs from CML mice 19 days post-transplantation (pooled data from n=3 BL/6 CML and n=4 *Ltbr*$^{-/-}$ CML). **(f)** Fold change in CFU capacity of *Light*$^{-/-}$ LSCs relative to BL/6 LSCs in serial re-plating, in absence or presence of 10 ng/ml recombinant LIGHT protein (BL/6 n=8, *Light*$^{-/-}$ n=7, for supplementation of rLIGHT: BL/6 n=7, *Light*$^{-/-}$ n=6). Data are shown as pooled data from two independent experiments. Data are presented as mean±SEM. Statistics: two-tailed *t*-test.

Supplementary Figure 8:

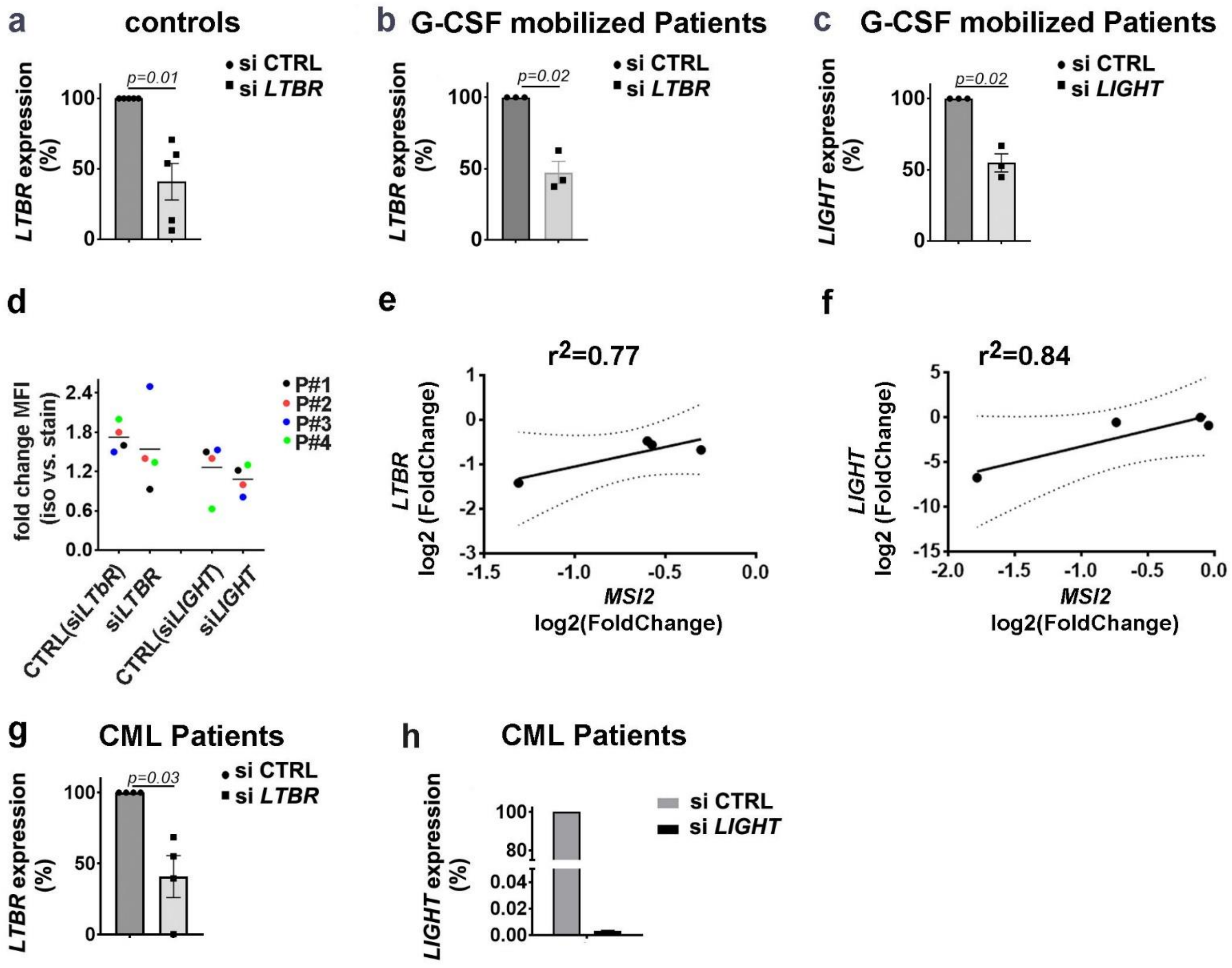

Supplementary Figure 8: LTβR and LIGHT siRNA knockdown efficiency.

(a) Relative mRNA expression of *LTBR* in si*LTBR* or siCTRL transfected HSPCs (CD45^{int}Lin^{-}CD34^{+}) from BM aspirates of untreated staging negative lymphoma patients (n=5). **(b)** Relative mRNA expression of *LTBR* in si*LTBR* or siCTRL transfected G-CSF mobilized PB HSPCs (n=3). **(c)** Relative mRNA expression of membranous *LIGHT* in si*LIGHT* or siCTRL transfected G-CSF mobilized PB HSPCs (n=3). **(d)** Fold change of surface LTβR or LIGHT protein in si*LTBR* or si*LIGHT* treated cells relative to siCTRL after colony formation. Bulk cells were FACS analyzed 14 days post-plating with human anti-LTβR and anti-LIGHT antibodies relative to isotype control. **(e)** Correlation analysis of *LTBR* (y-axis) and *MSI2* (x-axis) fold-change in gene expression from si*LTBR* and siCTRL transfected HSPCs. **(f)** Correlation analysis of *LIGHT* (y-axis) and *MSI2* (x-axis) fold-change in gene expression from si*LTBR* and siCTRL transfected HSPCs. **(g)** Relative mRNA expression of *LTBR* in si*LTBR* or siCTRL transfected HSPCs from CML patients (n=4 biological independent samples from n=5 CML patients). **(h)** Relative mRNA expression of membranous *LIGHT* in si*LIGHT* or siCTRL transfected HSPCs from CML patients (n=2). Data are presented as mean±SEM. Statistics: two-tailed *t*-test.

Supplementary Tables

Supplementary Table 1: Characteristics of G-CSF treated patients

Patient number	Diagnosis	Sex (f/m)	Age (yrs)
Patient 1 with G-CSF	Ewing sarcoma	m	19
Patient 2 with G-CSF	metastatic teratocarcinoma of the testis	m	47
Patient 3 with G-CSF	Diffuse Large B-cell lymphoma	m	29
Patient 4 with G-CSF	primitive neuroectodermal tumor	f	58
Patient 5 with G-CSF	Medulloblastoma	f	12

Supplementary Table 2: Characteristics of lymphoma patients

Patient number	Diagnosis	Sex (f/m)	Age (yrs)
Patient 1	Hodgkin-Lymphoma	m	32
Patient 2	Diffuse Large B-cell lymphoma	f	59
Patient 3	Diffuse Large B-cell lymphoma	m	61
Patient 4	Burkitt-Lymphoma	m	53
Patient 5	Non-Hodgkin-Lymphoma	m	80

Supplementary Table 3: Characteristics of CML patients

Patient number	Diagnosis	Sex (m/f)	Age (yrs)	Blood leukocytes [G/L]	Type of BCR-ABL1transcript	Blasts [%]	BCR-ABL1/ABL1 in % IS (International Scale)
1	CML in CP	m	72	174.3	b2a2	<5	40.9
2	CML in CP	m	65	179.7	b2a2	<5	31.6
3	CML in CP	m	21	217.1	b2a2	<5	28.14
4	CML in CP	m	76	43.3	b3a2	<5	34.3
5	CML in CP	m	28	166	b2a2	<5	35.7

Supplementary Table 4: Primer sequence of selected genes for fluidigm gene array

*selected as reference gene

Gene name	Forward primer (5'→3')	Len	Tm	Reverse primer (5'→3')	Len	Tm	Amp (bp)
*Actb**	AGATGACCCAGATCATGTTTGAG	23	60.64	GTACGACCAGAGGCATACAG	20	60.09	90
Akt1	CCATGAACGAGTTTGAGTACC	21	59.13	CAACCTCATCCTTGGCGA	18	59.79	140
Akt2	CCGCTATTATGCCATGAAGATCC	23	61.75	TTGAGGGCTGTAAGGAAGGG	20	61.71	122
Ap2a2	AACCTGCAGACTAAGCCC	18	59.38	GTCCCACCATACCTGAACTG	20	60.23	130
Bach1	TGTATCCATGACATCCGCAG	20	60.09	ACTTTGCAGCTTCTCGATTTCC	22	61.96	113
Bach2	GAGTTCATCCACGACATCC	19	58.09	TCTCGCACACCAGTTTCC	18	60.03	120
Bak1	CCAACAGCATCTTGGGTC	18	58.44	GAAGAGTTCGTAGGCATTCC	20	58.53	133
Bcl2	CGCAGAGATGTCCAGTCAG	19	60.32	AAGAAGGCCACAATCCTCC	19	59.78	116
Bcl2l1 (Bcl-x)	TTCGGGATGGAGTAAACTGG	20	59.58	GGTGGTCATTCAGATAGGTGG	21	60.36	140
Birc3	AACTCTCCAAGAAATCCAGCC	21	60.5	TATCGCCTTCACCTAAAGCA	20	59.28	126
Birc5	CTTCATCCACTGCCCTACC	19	59.94	TATGCTCCTCTATCGGGTTGTC	22	61.59	106
Bmi1	CAATGGCTCCAATGAAGACC	20	59.37	TACTTTCCGATCCAATCTGCTC	22	60.4	120
Casp3	TGGACTCTGGGATCTATCTGG	21	60.36	GAGATGACATTCCAGTGCTC	20	58.52	110
Casp8	TGATAAAGAGGCTCTGAGTAAGAC	24	59.88	AGTCTTTGTTCTTGTGGTCTG	21	58.56	130
Casp9	GCAGATATGGCATACACCC	19	58.21	GTGCTCAAGTTTGTCACGG	19	59.81	131
Ccnb1 (cyclin B1)	GTGAAGTGACTGGAAACATGAG	22	59.68	ACAACTGTTCTGCATGAACC	20	59.15	130
Ccnd1 (cyclin D1)	GAGCCCTTGAAGAAGAGCC	19	60.55	GTTCCATTTGCAGCAGCTC	19	60.25	150
Ccng1 (cyclin G1)	GCCATTTGAGAGGAGAAACGA	21	60.83	GCAAGGATAGATAGCGCCA	19	59.79	119
Ccng2 (cyclin G2)	CGACACGATGAAGGATTTGG	20	59.46	GCCTCCATCAAGATCAGCC	19	60.7	134
Cd99	CGAGTGACGACTTCAACCTG	20	61.13	GCCTGAGTCTCCGTGTG	17	59.79	148
Cdk4	AATGTTGTACGGCTGATGG	19	58.29	GTGCTTTGTCCAGGTATGTC	20	59.02	114
Cdk6	GAGTGCAGACCAGTGAGGA	19	61.39	ACACATCAAACAACCTGACCA	21	60.57	110
Cdkn1a (P21)	CCAGCCTGACAGATTTCTATCAC	23	61.26	ACACAGAGTGAGGGCTAAGG	20	61.4	143
Cdkn1b (P27)	AATTGGGTCTCAGGCAAACTC	21	61.05	AAGAAGAATCTTCTGCAGCAGG	22	61.13	130
Cdkn1c (P57)	CCAATCAGCCAGCAGAACAG	20	61.8	AGCTCCTCGTGGTCTACAG	19	60.85	113
Cdkn2a (P16)	GTGCTCTTTGTGTTCCGCT	19	61.36	GCTCTGCTCTTGGGATTGG	19	60.92	126
Cebpa	TCTGATTCTTGCCAAACTGAG	21	58.7	GCTAAGACCCACTACTACATACAC	24	60.48	184
Cebpb	AAGATGCGCAACCTGGAG	18	60.5	GCTTGAACAAGTTCCGCA	18	59.01	120
Cebpe	GTACCAAGTGGCACACTGC	19	61.73	TGCCTTCTTGCCCTTGTG	18	60.66	171
Ctnnb1	GCAAGTAGCTGATATTGACGG	21	59.34	CAAACTGCGTGGATGGGA	18	60.42	112
Cxcr4	CTGTAGAGCGAGTGTTGCC	19	60.91	GCAGGGTTCCTTGTTGGAG	19	61.08	108
Diablo (Smac)	GGTTCCTATTGCTCAGAAATCGG	23	61.81	GTGGTTTGAGACAGAAAGGTAGAG	24	61.32	110
Dnmt1	AGATTGAGACCACTGTTCCTCC	22	61.94	CTTGGCTTCGTCGTAACTCTC	21	61.21	115
Dnmt3a	ACACAGAAGCATATCCAGGAG	21	59.73	CCAGTACCCTCATAAAGTCCC	21	59.73	112
Dnmt3b	GAAGAATTTGAGCCACCCA	19	58.1	TCCTTGAGCACCAAGTACC	19	59.41	112
E2f1	CAGGGAAAGGTGTGAAATCTC	21	59.05	CCCAGTTCAGGTCAACGA	18	59.31	122

Eif2ak2	GCCTATCAGAAGCTGTTAAAGAG	23	59.38	ACTGGGAAACACCATTACTTGTC	23	61.27	120
Fgfr2	ATCTGCCTGGTCTTGGTC	18	59.05	TTTGGTTGGTGGCTCTTCTG	20	60.96	101
Foxp1	AGTGCGTCATAATCTTAGTCTCCA	24	61.71	TTAATAAGGGAAGGGTTACCACTG	24	60.31	134
Fzd3	ACGTGTCACACTTCCCTG	18	59.63	CATCACAATCTGGAAACCTACTG	23	59.56	124
Fzd7	GTTGACCTTTACCCTACCTTTGAC	24	61.57	ATATGAAGTAATCTGTCCTCCCGA	24	60.99	121
*Gapdh**	AGAACATCATCCCTGCATCC	20	60.01	TCATCATACTTGGCAGGTTTCTC	23	60.89	159
Gata1	TGAACTGTGGAGCAACGG	18	60.34	CTGACAATCATTCGCTTCTTGG	22	60.4	138
Gata2	CAGCAGTCTCTTCCATCCAG	20	60.3	GGCACCACAGTTGACAC	17	58.55	117
Gfi1	CCTTCAGCTCCCGAATTTCC	20	61.23	GATACTCTGAGTTCTCGTGCTG	22	60.72	117
Gli1	CTGAGACGCCATGTTCAATCC	21	61.83	ACCAGAAAGTCCTTCTGTTCC	21	60.08	120
Gsk3b	AAGTTCTACAGGACAAGCGA	20	59.14	ACCTCATCTTTCTTCTCACCAC	22	60.2	120
Hdac1	CATCTTTAAGCCAGTCATGTCC	22	59.55	GCGTGTCCTTTGATGGTC	18	58.86	128
Hes1	GTCATCAAAGCCTATCATGGAG	22	59.09	CGGGAGCTATCTTTCTTAAGTG	22	58.77	104
HoxB4	AAAGAGCCCGTCGTCTACC	19	61.82	CGATTGTAGTGAAACTCCTTCTCC	24	61.44	145
Il1rl1	CCCTGATTATTTGATGTACTCGAC	24	59.52	AGAGCTTTGCAGTTCTTAAACC	22	59.61	122
Il4	CAGCAACGAAGAACACCAC	19	59.81	GCAGCTCCATGAGAACAC	18	58.54	125
Il7	TGATCAGCATCGATGAATTGGAC	23	61.57	AAGCAGCTTCCTTTGTATCATCAC	24	61.93	110
Il33	GGCAAAGTTCAGCAGCAC	18	60.11	CAGAACGGAGTCTCATGCAG	20	60.93	112
Igf1r	CTTCCTGTGAAAGTGATGTTCTC	23	59.76	GTGCCTCCTTGTAGTAAACTG	21	58.78	134
Irf8	GTATGACCAAGAGGAGCCC	19	59.64	ATCTGGGAGAAAGCTGAATGG	21	60.29	144
Jag1	CTTGCCATTCCCGTGACAG	19	61.51	GTTGTTCCTTCCCAGCCAC	19	61.38	114
Jag2	ATTGATGAGTGCCAGTCCT	19	58.77	CGTGAATATGACCACTTCCTG	21	58.93	125
Jun	CCAAGAACTCGGACCTTCTC	20	60.23	GTGATGTGCCCATTGCTG	18	59.56	102
Kif3a	CAGAGGTTAGAGGTTAAAGAAAGG	24	59.03	GCTCGTTCATATTAGTCGCAC	21	59.69	147
Kit	GTGTTTGTTAGAGATCCTGCCA	22	60.8	TCGATGAGGGAATAATTGGACAC	23	60.7	124
Klf1	GACTTCCTCAAGTGGTGGC	19	60.77	GTCCCTCTCATCGTCCTCTC	20	61.31	128
Lef1	ACCCTCCTACTCCAGTTACTC	21	60.22	GGGCACTTTATTTGATGTCCTC	22	59.81	114
Lta	CTTTCCTGCCTTCGACTG	18	58.15	TGTGGAGAACCTGCTGTG	18	59.62	162
Ltb	GATGACCATCCTGTCTCCA	19	58.55	CATCCAAGCGCCTATGAG	18	58.07	126
LTβR	CCCATACCAGATGTGAGATCC	21	59.87	GGTGAAGAGCAGAAAGAGGAC	21	60.83	145
Maml1	ATACCTCAGCAGCCAGCA	18	61.16	TTGTTTCTCCTGCTCAGCC	19	60.4	147
Msi2	CGCTATGGAGGCAAATGGG	19	61.37	TAAGGCTATCTGGTGAGGTCTG	22	61.21	115
Myc	CAGTGGTCTTTCCCTACCC	19	59.48	TCTTGCTCTTCTTCAGAGTCG	21	59.94	192
Nfkb1	CTGAGAAGGAAACTGAAGGTG	21	58.71	GAAAGAGGTTATCCTGAAATCCC	23	59.05	125
Nfkb2	CATCCATGACAGCAAGTCTCC	21	61.24	ATCATCCTCATAGAACCGAACC	22	59.87	150
Notch1	CAGACCAACACGCAGTACC	19	61.13	CGTCAATGCCTCGCTTCTG	19	61.92	108
Notch2	CGCATTAAACAAGATTCTCAGGG	23	60.44	AATATCTTAGAGCCTATCACCTCC	24	59.25	145
Numb	GAGAAAGAAAGACGTTTATGTCCC	24	60.12	GTGGCCGAGGTACTTAACTG	20	60.59	111
Pafah1b1 (Lis1)	GATGCTACAATTAAGGTGTGGGA	23	60.89	TAATCGTCATATCTGCTGAACAGG	24	60.78	138
Pik3ca	GAGAGTTTGAGAGGTTTCAGG	21	58.43	AAGATTGTAGTTCTGGCATTCC	22	58.88	122
Ptch1	TGTTCCAGTTAATGACTCCCA	21	59.16	ACTTTGATGAACCACCTCCAC	21	60.7	142
Rela	TCGAATCTCCCTGGTCAC	18	58.42	GTTCTGGAAGCTATGGATACTG	22	58.5	129

Relb	**CGACAAGAAGTCCACCAACAC**	**21**	**61.9**	**TGTGCTGAACACCACGG**	**17**	**60.28**	**138**
Runx1	**GAGGTACTAGCTGACCACCC**	**20**	**61.53**	**TGCCACCACCTTGAAAGC**	**18**	**60.98**	**122**
Satb1	**TGATCTCCTCCATTGTGAACAG**	**22**	**60.27**	**CTATCCATCTCAACCATCATATCC**	**24**	**58.59**	**120**
Sirt1	**GCACTAATTCCAAGTTCTATACCC**	**24**	**59.46**	**ACCACCTAGCCTATGACAC**	**19**	**58.55**	**140**
Smo	**CACCTCCTTCAAAGCCCTG**	**19**	**60.78**	**AGTCTCCATCTACCTGAGCC**	**20**	**60.6**	**133**
Spi1 (PU.1)	**CCATAGCGATCACTACTGGG**	**20**	**59.87**	**CATGTGGCGATAGAGCTG**	**18**	**58.08**	**132**
Tet2	**AGAACACTCATGGAAGAAAGG**	**21**	**58.06**	**CCGATATACCCATTTAGCAATAGG**	**24**	**59.15**	**122**
Tnfsf14 (LIGHT)	**GAGACATAGTAGCTCATCTGCC**	**22**	**60.27**	**CGTTGGCTCCTGTAAGATGTG**	**21**	**61.5**	**110**
Tnik	**TGATTTCAAGAGTGACTTGTGG**	**22**	**59.02**	**ACTTCTTAGACTTGAGCCGAG**	**21**	**59.67**	**142**
Traf2	**AGAGTAGTTCGGCCTTTCCA**	**20**	**61.04**	**TCTCGTATTCTTTCAAGGTCCC**	**22**	**60.01**	**110**
Traf3	**CGGCACAGAATTTCAGTTTCC**	**21**	**60.55**	**TTGTAGCCTCCTTGCTCC**	**18**	**59.05**	**140**
Trp53	**ATGTGCACGTACTCTCCTC**	**19**	**59.27**	**TGCTGTGACTTCTTGTAGATGG**	**22**	**60.53**	**136**
Wisp2	**TGTGCCTCTTCGAAGAGGA**	**19**	**60.71**	**GTGAAACCACCGTCATCAC**	**19**	**59.21**	**117**

References

1. Untergasser A, Nijveen H, Rao X, Bisseling T, Geurts R, Leunissen JA. Primer3Plus, an enhanced web interface to Primer3. *Nucleic Acids Res* **35**, W71-74 (2007).

2. Livak KJ, Schmittgen TD. Analysis of relative gene expression data using real-time quantitative PCR and the 2(-Delta Delta C(T)) Method. *Methods* **25**, 402-408 (2001).
