# Supplementary material for: LIGHT/LTβR signaling regulates self-renewal and differentiation of hematopoietic and leukemia stem cells
Source: Nat Commun. 2021 Feb 16;12:1065. doi: 10.1038/s41467-021-21317-x (PMC7887212; doi:10.1038/s41467-021-21317-x)
Supplement: Supplementary file 2 — Reporting Summary [file 41467_2021_21317_MOESM2_ESM.pdf]

nature research

Corresponding author(s): Adrian F. Ochsenbein

Last updated by author(s): 20/11/2020

Reporting Summary

Nature Research wishes to improve the reproducibility of the work that we publish. This form provides structure for consistency and transparency in reporting. For further information on Nature Research policies, see our Editorial Policies and the Editorial Policy Checklist.

Statistics

For all statistical analyses, confirm that the following items are present in the figure legend, table legend, main text, or Methods section.

n/a	Confirmed	
☐	☒	The exact sample size (*n*) for each experimental group/condition, given as a discrete number and unit of measurement
☐	☒	A statement on whether measurements were taken from distinct samples or whether the same sample was measured repeatedly
☐	☒	The statistical test(s) used AND whether they are one- or two-sided<br>*Only common tests should be described solely by name; describe more complex techniques in the Methods section.*
☒	☐	A description of all covariates tested
☒	☐	A description of any assumptions or corrections, such as tests of normality and adjustment for multiple comparisons
☐	☒	A full description of the statistical parameters including central tendency (e.g. means) or other basic estimates (e.g. regression coefficient) AND variation (e.g. standard deviation) or associated estimates of uncertainty (e.g. confidence intervals)
☐	☐	For null hypothesis testing, the test statistic (e.g. *F*, *t*, *r*) with confidence intervals, effect sizes, degrees of freedom and *P* value noted<br>*Give P values as exact values whenever suitable.*
☐	☐	For Bayesian analysis, information on the choice of priors and Markov chain Monte Carlo settings
☒	☐	For hierarchical and complex designs, identification of the appropriate level for tests and full reporting of outcomes
☒	☐	Estimates of effect sizes (e.g. Cohen's *d*, Pearson's *r*), indicating how they were calculated

Our web collection on statistics for biologists contains articles on many of the points above.

Software and code

Policy information about availability of computer code

Data collection	no software was used
Data analysis	Flow cytometric data were analyzed using FlowJo(V.10.0.8,TreeStar, Inc.)<br>software Kaluza Analysis software (v.2.1 Beckman Coulter)<br>ImageStreamX ® System Software INSPIRE®200.1.681.0<br>ImageStreamX® analysis was performed using Amnis IDEAS® software IFX328<br>RNA-seq data Array Star softwarev16<br>Seq Man NGen software v.16<br>Ariadne Genomic Pathway Studio® database (www.elsevier.com)<br>Gene set enrichment analysis software v3.0 Illumina RTA version 2.4.11<br>Basecalling Version bcl2fastq2.20.0.422<br>Graph Prism Software 7.0<br>Graph Prism Software 8.0

For manuscripts utilizing custom algorithms or software that are central to the research but not yet described in published literature, software must be made available to editors and reviewers. We strongly encourage code deposition in a community repository (e.g. GitHub). See the Nature Research guidelines for submitting code & software for further information.

Data

Policy information about availability of data

All manuscripts must include a data availability statement. This statement should provide the following information, where applicable:

- Accession codes, unique identifiers, or web links for publicly available datasets
- A list of figures that have associated raw data
- A description of any restrictions on data availability

Data are available in the NCBI Data GEO baseaccession code: GSE141206 (https://www.ncbi.nlm.nih.gov/geo/query/acc.cgi?acc=GSE141206)
Data are available under accession number GSE3271946 (https://www.ncbi.nlm.nih.gov/geo/query/acc.cgi?acc=GSE32719) and
GSE1167547 (https://www.ncbi.nlm.nih.gov/geo/query/acc.cgi?acc=GSE11675)

Field-specific reporting

Please select the one below that is the best fit for your research. If you are not sure, read the appropriate sections before making your selection.

☐ Life sciences ☐ Behavioural & social sciences ☐ Ecological, evolutionary & environmental sciences

For a reference copy of the document with all sections, see nature.com/documents/nr-reporting-summary-flat.pdf

Life sciences study design

All studies must disclose on these points even when the disclosure is negative.

Sample size	Sample size and group size was determined based on the historical practical experience in the laboratory during the last 10 years.
Data exclusions	no data were excluded
Replication	Experiments were performed two or more times, independently from each other and data could successfully reproduced.
Randomization	mice were sex and aged matched and randomized whenever possible. Human samples were allocated based the availability of sufficient frozen cells. Experimental groups were controlled internally within each experiment.
Blinding	Experiments were performed in an unblinded manner.

Behavioural & social sciences study design

All studies must disclose on these points even when the disclosure is negative.

Study description	*Briefly describe the study type including whether data are quantitative, qualitative, or mixed-methods (e.g. qualitative cross-sectional, quantitative experimental, mixed-methods case study).*
Research sample	*State the research sample (e.g. Harvard university undergraduates, villagers in rural India) and provide relevant demographic information (e.g. age, sex) and indicate whether the sample is representative. Provide a rationale for the study sample chosen. For studies involving existing datasets, please describe the dataset and source.*
Sampling strategy	*Describe the sampling procedure (e.g. random, snowball, stratified, convenience). Describe the statistical methods that were used to predetermine sample size OR if no sample-size calculation was performed, describe how sample sizes were chosen and provide a rationale for why these sample sizes are sufficient. For qualitative data, please indicate whether data saturation was considered, and what criteria were used to decide that no further sampling was needed.*
Data collection	*Provide details about the data collection procedure, including the instruments or devices used to record the data (e.g. pen and paper, computer, eye tracker, video or audio equipment) whether anyone was present besides the participant(s) and the researcher, and whether the researcher was blind to experimental condition and/or the study hypothesis during data collection.*
Timing	*Indicate the start and stop dates of data collection. If there is a gap between collection periods, state the dates for each sample cohort.*
Data exclusions	*If no data were excluded from the analyses, state so OR if data were excluded, provide the exact number of exclusions and the rationale behind them, indicating whether exclusion criteria were pre-established.*
Non-participation	*State how many participants dropped out/declined participation and the reason(s) given OR provide response rate OR state that no participants dropped out/declined participation.*
Randomization	*If participants were not allocated into experimental groups, state so OR describe how participants were allocated to groups, and if allocation was not random, describe how covariates were controlled.*

Ecological, evolutionary & environmental sciences study design

All studies must disclose on these points even when the disclosure is negative.

Study description	*Briefly describe the study. For quantitative data include treatment factors and interactions, design structure (e.g. factorial, nested, hierarchical), nature and number of experimental units and replicates.*
Research sample	*Describe the research sample (e.g. a group of tagged Passer domesticus, all Stenocereus thurberi within Organ Pipe Cactus National Monument), and provide a rationale for the sample choice. When relevant, describe the organism taxa, source, sex, age range and any manipulations. State what population the sample is meant to represent when applicable. For studies involving existing datasets, describe the data and its source.*
Sampling strategy	*Note the sampling procedure. Describe the statistical methods that were used to predetermine sample size OR if no sample-size calculation was performed, describe how sample sizes were chosen and provide a rationale for why these sample sizes are sufficient.*
Data collection	*Describe the data collection procedure, including who recorded the data and how.*
Timing and spatial scale	*Indicate the start and stop dates of data collection, noting the frequency and periodicity of sampling and providing a rationale for these choices. If there is a gap between collection periods, state the dates for each sample cohort. Specify the spatial scale from which the data are taken*
Data exclusions	*If no data were excluded from the analyses, state so OR if data were excluded, describe the exclusions and the rationale behind them, indicating whether exclusion criteria were pre-established.*
Reproducibility	*Describe the measures taken to verify the reproducibility of experimental findings. For each experiment, note whether any attempts to repeat the experiment failed OR state that all attempts to repeat the experiment were successful.*
Randomization	*Describe how samples/organisms/participants were allocated into groups. If allocation was not random, describe how covariates were controlled. If this is not relevant to your study, explain why.*
Blinding	*Describe the extent of blinding used during data acquisition and analysis. If blinding was not possible, describe why OR explain why blinding was not relevant to your study.*

Did the study involve field work? ☐ Yes ☐ No

Field work, collection and transport

Field conditions	*Describe the study conditions for field work, providing relevant parameters (e.g. temperature, rainfall).*
Location	*State the location of the sampling or experiment, providing relevant parameters (e.g. latitude and longitude, elevation, water depth).*
Access & import/export	*Describe the efforts you have made to access habitats and to collect and import/export your samples in a responsible manner and in compliance with local, national and international laws, noting any permits that were obtained (give the name of the issuing authority, the date of issue, and any identifying information).*
Disturbance	*Describe any disturbance caused by the study and how it was minimized.*

Reporting for specific materials, systems and methods

We require information from authors about some types of materials, experimental systems and methods used in many studies. Here, indicate whether each material, system or method listed is relevant to your study. If you are not sure if a list item applies to your research, read the appropriate section before selecting a response.

Materials & experimental systems

n/a	Involved in the study	
☐	☒	Antibodies
☒	☐	Eukaryotic cell lines
☒	☐	Palaeontology and archaeology
☐	☒	Animals and other organisms
☐	☒	Human research participants
☒	☐	Clinical data
☒	☐	Dual use research of concern

Methods

n/a	Involved in the study	
☒	☐	ChIP-seq
☐	☒	Flow cytometry
☒	☐	MRI-based neuroimaging

Antibodies

Antibodies used

anti-Mouse :
αCD31-F ITC (clone:390), Biolegend, San Diego, United States,Cat# 102405, 1:100
αCD51-PE (clone:RMV -7) Biolegend San Diego, United States,Cat#104105, 1:100
αCD51-Alexa 647 (clone:RMV -7) Bio-Rad AbDSerotec, Oxford, United Kingdom, Cat#MCA2461A647T 1:25
α Ter119-biot(Ter-119) Biolegend, San Diego, United States,Cat#116203,1:300
α CD19-biot (6D5) Biolegend San Diego, United States,Cat#115503,1:300
α CD3-biot(145-2C1) Biolegend San Diego, United States,Cat#100304, 1:300
α Gr1-biot(RB6-8C5) Biolegend San Diego, United States,Cat#108404,1:300
αLy-6A/ E- PerCP-C y5.5 (clone:D7) Biolegend San Diego, United States,Cat#108122S, 1:600
αCD117-APC-Cy7 (clone:2B8) , Biolegend San Diego , United States,Cat#105826, 1:300,
αCD48-PE-Cy7 (clone: HM 48-1), Biolegend, San Diego, United States,Cat#103424, 1:100
αCD150-APC (clone:TC1512F12 .2), Biolegend, San Diego, United States,Cat#115909, 1:400,
αCD150-Alexa Fluor 488 (clone:TC1512 F12 .2), Biolegend, San Diego, United States,Cat#115915, 1:400,
αLy-6A/ E-APC (clone:D7) eBioscience, Waltham, MA, United States, Cat#l7-5981-82, 1:600
αCD16/CD32-PE-Cy7 (clone:93), Biolegend, San Diego, United Stat es,Cat#101318, 1:400
αCD34-eFluor-450 (clone:RAM34), eBioscience, Waltham, MA, United States, Cat# 48-0341-82, 1:100
αCD135-PE (clone:A2F10), Biolegend , San Diego, United States,Cat#135306, 1:100
αCD127-FITC (clone:A7R34), Biolegend, San Diego, United States,Cat#135008, 1:100
αCD45.l- PerCP -Cy5.5 (clone:A20), Biolegend, San Diego, United States,Cat#l10728, 1:200
αLTβR-PE (clone:ebio3C8), eBioscience, Waltham, MA, United States, Cat#13-5671-82, 1:20
αLy-6C-PerCP-Cy5.5 (clone:HKl.4), Biolegend, San Diego, United States,Cat# 128011, 1:100
αCD11b-PE-Cy7, (clone:M l /70), Biolegend, San Diego, United States,Cat#101216, 1:200
αCD8a-FITC, (clone:53-6.7), Biolegend, San Diego, United States,Cat#100705, 1:600
αCD8a-APC(clone:53-6.7), Biolegend , San Diego, United States,Cat#100711, 1:600
αCD4-FITC (clone:GKl. 5), Biole gend, San Diego, United States,Cat#100405, 1:800
αCD90.l-APC (clone:OX-7), Biole gend, San Diego, United States,Cat#202526, 1:100
αCD90.2-APC (clone:30-H12), Biolegend, San Diego, United States,Cat#l05311, 1:100
Annexin-V-Pacific-Blue, Biolegend, San Diego, United States,Cat# 640917, 1:100
Annexin V-Alex-647, Biolegend, San Diego, United States,Cat# 640911, 1:100
Annexin V-PE, Biolegend, San Diego, United States,Cat# 640907, 1:100
Streptavidin V500, BD Bioscience , San Jose, CA, United States, Cat#561419, 1:1000
Streptavidin PE, Biolegend, San Diego, United States,Cat#405203, 1:1000
αLy-6G-Pacific-Blue (clone:1A8), Biolegend, San Diego, United States,Cat# 127611 , 1:500
αCD45.2-Alexa-700 (clone:104), Biolegend, San Diego, United States,Cat#109822, 1:100
Ki 67-PE (clone:16A8), ebioscience, Walth am, MA, United States, Cat#12-4321-80, 1:100
rat lgG2a,κPE (clon e:RTK2 758), Biolegend, San Diego, United States, Cat#400507
αCD45-PerCP-Cy5.5 (clone:30-Fll)Biolegend, San Diego, United States,Cat# 103131, 1:200
αCD45-PE-Cy7 (clone:30-Fll)Biolegend, San Diego, United States,Cat# 103113, 1:200
αCD45-APC (clone:30-Fll)Biolegend, San Diego, United States,Cat# 103111, 1:200
α phospho lkb-α (clone:14D4), Cell signaling Technology, Massachusetts, United States,Cat# 2859, 1:100
αNFkBp65 (D14E12)Cell signaling Technology, Massachusetts, United States,Cat# 8242, 1:100
α phospho Ikk-a/b (16A6)Cell signaling Technology, Massachusetts, United States,Cat# 2697, 1:500
α Ikb-α (clone:44D4), Cell signaling Technology, Massachusetts, United States,Cat# 4812, 1:100
α rabbit lgG(H +L), F(ab)2 Fragment-Alexa Fluor 647, Cell signaling Technology, Massachusetts, United States,Cat# 4412 1:800

anti-Human:
αCD90-PeCP-Cy5.5 (clone:5E10), Biolegend, San Diego, United States,Cat# 328117, 1:100
αCD34-APC (clone:561), Biole gend, San Diego, United States,Cat# 343608, 1:80
αLTβR-PE (clone:31G4D8), Biolegend, San Diego, United States,Cat# 322008, 1:80
αCD38-APC (clone:HIT2), Biole gend, San Diego, United States,Cat# 303510, 1:50
αCD38-PE-Cy7 (clone:H IT2), Biolegend , San Diego, United States,Cat#303515, 1:50
mouse-lgG2b,κ(clone:MPC-11), Biolegend, San Diego, United States,Cat# 400313
αCD2-biot in (clone:RPA2.10), Biolegend, San Diego, United States,Cat# 300204, 1:100
αD3Ɛbiotin (clone:OKT3), Biolegend, San Diego, United States,Cat# 317320, 1:100
αCD14-biot in (clone:HCD1 4), Biolegend, San Diego, United States,Cat# 325624, 1:100
αD16-biot in (clone:3G8), Biolegend, San Diego, United States,Cat# 302004, 1:100
αCD19-biot in (clone:HIB19), Biolegend, San Diego, United States,Cat# 302204, 1:100
αCD56-biot in (c lo n e:HCD56), Biolegend, San Diego, United States,Cat# 318320, 1:100
αCD235a-b iotin (clone:HIR2), Bio legend, San Diego, United States,Cat# 306618, 1:100
αCD45-V500C (clone:2Dl), BD Bioscience, San Jose, CA, United States, Cat# 647449 1:50
αCD45-Pacific Blue (clone:2Dl), Biolegend, San Diego, United States, Cat# 368539 1:100
Streptavidin-FITC, Biolegend, San Diego, United States,Cat# 405201, 1:1000

lmageStream:
Rabbit-anti-Numb (ab4147) Abcam, Cambridge, MA, United States, Cat#ab4147, 1:20

	Mouse-anti a-tubulin (ab7291) Abcam, Cambridge, MA, United States, Cat#ab7291, 1:400<br>Goat-anti-rabbit Alexa Fluor 568, Abcam, Cambridge, MA, United States, Cat#ab7175695, 1:400<br>Goat-anti mouse Alexa 647, Abcam, Cambridge, MA, United States, Cat#ab150115, 1:2000<br>Donkey anti-goat, Abcam, Cambridge, MA, United States, Cat#ab1175704, 1:400
Validation	antibodies were validated based on manufacturer´s information on appropriate cell lines, splenocytes (isolated from C57/Bl6 mice)or human PBMCs by appropriate titration based on manufacturer´s information. Optimal antibody concentration was used, depicted in this report summery and in the method section of the manuscript.

Animals and other organisms

Policy information about studies involving animals; ARRIVE guidelines recommended for reporting animal research

Laboratory animals	C57BL/6 mice , male and female, Charles River<br>Ly5.1, mice, male and female, Charles River<br>Ltbr-/- mice, BL/6 (CD45.2) background, male and female (in house)<br>Light-/- mice, BL/6 (CD45.2)background, male and female (in house)<br>Light-/- BL/6 (CD45.1) background, male and female (in house)<br>Ltbr-/-/Light-/-, double knockout mice, BL/6 (CD45.2) background, male and female (in house)<br>Knockout strains are homozygote mutant mice, generated by intragenic deletions.<br>Intercrosses were validated by genotyping PCR.<br>Offspring mice from Ly5.1xBL/6, mice, male and female<br>C57BL/6-Tg(UBC-GFP)30Scha/J, mice, male and female<br>Animal housing was performed under standard conditions, at a temperature of 22±2°C with 50±15% of humidity level and a light/dark cycle of 12h<br><br>all mice aged 6-13 weeks were used for experiments
Wild animals	no wild animals were used
Field-collected samples	no field collection samples were used
Ethics oversight	Animal experiments were approved by the local experimental animal committee of the Canton of Bern and performed according to Swiss laws for animal protection.

Note that full information on the approval of the study protocol must also be provided in the manuscript.

Human research participants

Policy information about studies involving human research participants

Population characteristics	Patient characterization is depicted in the Supplementary tables 1-3.<br>G-CSF patients (male and female) were diagnosed with following diseases: Ewing sarcoma,metastatic teratocarcinoma of the testis, Diffuse Large B-cell lymphoma, primitive neuroectodermal tumor Medulloblastoma, age ranges between 12 and 58 years.<br>Lymphoma patients (male and female) were diagnosed with following diseases: Hodgkin-Lymphoma, Diffuse Large B-cell lymphoma , Burkitt-Lymphoma, Non-Hodgkin-Lymphoma, age range between 32 and 80 years<br>CML patients (male) were diagnosed as CML in chronic phase and were between 21 and 76 years old
Recruitment	Samples of patients with written informed consent are obtained from the Biobank in Bern.
Ethics oversight	Patient samples were collected at the University Hospital of Bern after written informed consent. Analysis of samples was approved by the local ethical committee of the Canton of Bern (KEK122/14).

Note that full information on the approval of the study protocol must also be provided in the manuscript.

Flow Cytometry

Plots

Confirm that:

- [x] The axis labels state the marker and fluorochrome used (e.g. CD4-FITC).
- [x] The axis scales are clearly visible. Include numbers along axes only for bottom left plot of group (a 'group' is an analysis of identical markers).
- [x] All plots are contour plots with outliers or pseudocolor plots.
- [x] A numerical value for number of cells or percentage (with statistics) is provided.

Methodology

Sample preparation	Surface staining:<br>Ex vivo isolated cells from BM or blood were ACK lysed and surface stained with appropriate antibodies for minimum 30 min at 4°C in FACS buffer or appropriate buffer. For intracellular stainings, ex vivo isolated cells were first stained with surface antibodies for 30 min at 4°C. Subsequently cells were permeabilized with eBioscience'" Foxp3 / Transcription Factor Staining Buffer Set (ebioscience, Waltham, MA, united States), washed in FOXP3 wash buffer and intracellular stained with appropriate antibodies in FOXP3 wash buffer for 30 min at 4°C.<br>Intracellular staining for members of the NFkB pathway:<br>Previously surface-stained cells were fixed in 4% PFA followed by ice-cold methanol permeabilisation, according to manufacturer's protocol. Subsequently, cells were intracellular stained in 0.5% BSA PBS buffer.
Instrument	The BD LSRFortessa'" or the BD LSRII SORP were used for data acquisition. FACS-sorting was performed by using BD FACSAria Ill
Software	FACS analysis was performed by using FlowJo(V.10.0.8,TreeStar, Inc.) software and<br>Kaluza Analysis software (v.2.1 Beckman Coulter)
Cell population abundance	A minimum of 200.000 cells/ sample were acquired for flow cytometry. A minimum of 5.000 cells/sample were acquired for ST/ LT-HSCs cell cycle analysis and analysis of members of the NFkB pathway.
Gating strategy	FSC/SSC (cells of interest)> FSC-H/FSC-A (doublet exclusion)>SSC/Annexin -V (Live/dead separation)> SSC/Lineage (Lineage negative cells)> CDll7/sca-1 (LSKs)> CD150/CD48 (primititive HSCs and MPPs)

- [x] Tick this box to confirm that a figure exemplifying the gating strategy is provided in the Supplementary Information.
