# Supplementary material for: LIGHT/LTβR signaling regulates self-renewal and differentiation of hematopoietic and leukemia stem cells
Source: Nat Commun. 2021 Feb 16;12:1065. doi: 10.1038/s41467-021-21317-x (PMC7887212; doi:10.1038/s41467-021-21317-x)
Supplement: Supplementary file 3 — Description of Additional Supplementary Files [file 41467_2021_21317_MOESM3_ESM.pdf]

Description of Additional Supplementary Files

Supplementary Data 1:

Differentially expressed genes in LTbR- deficient HSCs relative to LTbR proficient HSCs

FACS-purified Ltbr-/- and Ly5.1 LT/ST-HSCS from chimeras six weeks after primary transplantation (n=6, pooled to 3 biological replicates) were used for RNA-seq analysis. Supplementary data 1 includes all 227 differentially expressed genes.
